# Genes Influenced by the Non-Muscle Isoform of Myosin Light Chain Kinase Impact Human Cancer Prognosis

**DOI:** 10.1371/journal.pone.0094325

**Published:** 2014-04-08

**Authors:** Tong Zhou, Ting Wang, Joe G. N. Garcia

**Affiliations:** Arizona Respiratory Center and Department of Medicine, The University of Arizona, Tucson, Arizona, United States of America; University of Chicago, Department of Medicine, United States of America

## Abstract

The multifunctional non-muscle isoform of myosin light chain kinase (nmMLCK) is critical to the rapid dynamic coordination of the cytoskeleton involved in cancer cell proliferation and migration. We identified 45 nmMLCK-influenced genes by bioinformatic filtering of genome–wide expression in wild type and nmMLCK knockout (KO) mice exposed to preclinical models of murine acute inflammatory lung injury, pathologies that are well established to include nmMLCK as an essential participant. To determine whether these nmMLCK-influenced genes were relevant to human cancers, the 45 mouse genes were matched to 38 distinct human orthologs (M38 signature) (GeneCards definition) and underwent Kaplan-Meier survival analysis in training and validation cohorts. These studies revealed that in training cohorts, the M38 signature successfully identified cancer patients with poor overall survival in breast cancer (*P*<0.001), colon cancer (*P*<0.001), glioma (*P*<0.001), and lung cancer (*P*<0.001). In validation cohorts, the M38 signature demonstrated significantly reduced overall survival for high-score patients of breast cancer (*P* = 0.002), colon cancer (*P* = 0.035), glioma (*P* = 0.023), and lung cancer (*P* = 0.023). The association between M38 risk score and overall survival was confirmed by univariate Cox proportional hazard analysis of overall survival in the both training and validation cohorts. This study, providing a novel prognostic cancer gene signature derived from a murine model of nmMLCK-associated lung inflammation, strongly supports nmMLCK-involved pathways in tumor growth and progression in human cancers and nmMLCK as an attractive candidate molecular target in both inflammatory and neoplastic processes.

## Introduction

Cancer cell proliferation and migration require rapid dynamic regulation of the cytoskeleton, which is controlled by series of cytoskeleton regulatory proteins, in which myosin light chain kinase (MLCK) is a critical participant [1], [2]. In addition, endothelial cell paracellular extravasation and diapedesis by tumor cells is an essential step for malignant tumor metastasis and significantly influenced the activity of MLCK [3], [4]. Although still underestimated, MLCK started to be considered as a novel functional protein in cancer pathogenesis (initiation, proliferation, migration, and metastasis) [5], [6], [7]. This is especially true with the more widely expressed non-muscle isoform (nmMLCK). Non-muscle myosin light chain kinase or nmMLCK is centrally involved in driving rearrangement of the cytoskeleton, which regulates vascular endothelial barrier function, angiogenesis, endothelial cell apoptosis, and leukocytic diapedesis [8]. *In vivo* studies implicated nmMLCK as an attractive target for ameliorating the adverse effects of dysregulated lung inflammation, including extravasation of inflammatory leukocytes [9], [10], similar with the procedure of cancer cell metastasis to lung tissues [11]. Deletion or silencing of nmMLCK produced greater protection against acute lung injury (ALI) and ventilator-induced lung injury (VILI) and significantly decreased alveolar and vascular permeability and lung inflammation [9].

Recently, we reported that endothelial inflammation is a key mediator of tumor growth and progression [12], supported by the fact that a molecular signature reflective of the endothelial inflammatory gene expression is predictive of clinical outcome in multiple types of human cancer [12]. As nmMLCK plays a central role in regulation of endothelial cytoskeleton and endothelial inflammation, we would hypothesize that nmMLCK-related cellular signaling actively participate in the tumor progression and metastasis, although little is known regarding the effect of nmMLCK on the pathogenesis of tumor and its influence on the prognosis of human cancers.

In this present study, we would like to use nmMLCK-associated gene network (nmMLCK-deregulated gene sets) to establish a novel methodology for human cancer prognoses, by using a computational biology approach.

The purpose of this study is two-fold. The first was to identify the genes potentially regulated by nmMLCK. The second was to develop a prognostic cancer gene signature derived from the nmMLCK-associated genes. Using an experimental murine model of lung injury induced by mechanical ventilation with increased tidal volumes (the VILI model), we characterized the top differentially expressed genes between VILI-challenged wild-type (WT) mice and nmMLCK knockout (KO) mice. The mouse genes mediated by nmMLCK expression were identified. We matched the nmMLCK-mediated mouse genes to their human orthologs, which formed the basis of a multivariate molecular predictor of overall survival in several human cancers, including lung cancer, breast cancer, colon cancer, and glioma. This molecular signature predicted outcome independently of, but cooperatively with, standard clinical and pathological prognostic factors including patient age, lymph node involvement, tumor size, tumor grade, and so on.

## Materials and Methods

### Gene expression data

Microarray data of lung RNA from WT control, VILI-exposed WT, and VILI-exposed nmMLCK KO mice were obtained from NCBI GEO database (GSE14525) [9]. We used this dataset to filter out the nmMLCK-mediated mouse genes.

The gene expression datasets representing human cancers were downloaded from publicly available repositories. These datasets were chosen based on the availability of clinical survival data and the large size of samples. For each tumor type, training and validation cohorts were constructed. The dataset for breast cancer (n = 295) was available from http://bioinformatics.nki.nl/data.php (Netherlands Cancer Institute, Computational Cancer Biology Data Repository) [13]. The breast cancer patients were randomly separated into two parts (1/2 for training and 1/2 for validation). For colon cancer, we downloaded two datasets from a single study [14]. One dataset was used as training cohort (n = 177; GSE17536) and the other one was used for validation (n = 55; GSE17537). For glioma, distinct datasets from two different studies were obtained for training (n = 77; GSE4271) [15] and validation (n = 50; http://www.broadinstitute.org/cgi-bin/ca?ncer/datasets.cgi) [16]. Lastly, we obtained three datasets (n = 359) for lung cancer which were available from a single study [17]. Two datasets were combined as training cohort (n = 161) and the other one was used as validation cohort (n = 178). The CEL files for the study are available at https://caarraydb.nci.nih.gov/caarray/publicExperimentDetailAction.do?expId=1015945236141280.

### Statistical analysis

SAM (Significance Analysis of Microarrays) [18], implemented in the *samr* library of the R Statistical Package [19], was used to compare log_2_-transformed gene expression levels between WT control, VILI-exposed WT, and VILI-exposed nmMLCK KO mice. False discovery rate (FDR) was controlled using the q-value method [20]. Transcripts with a fold-change greater than 2 and FDR less than 10% were deemed differentially expressed. We searched for any enriched Kyoto Encyclopedia of Genes and Genomes (KEGG) [21] physiological pathways among the differential genes relative to the final analysis set using the NIH/DAVID [22], [23]. Hierarchical clustering via complete linkage rule with Euclidean distance metric was applied to visualize gene expression differences, using *gplots* library of R Statistical Package [19].

For each cancer training/validation dataset, we normalized the gene expression level into the scale of [−1, 1] by POE (probability of expression) algorithm [24], [25] implemented in the *metaArray* library of the R Statistical Package [19]. Based on the gene expression and clinical outcome data from the training dataset, we can assign a Wald statistic generated by univariate Cox proportional-hazard regression to each gene as a weight. A risk score was calculated for each patient using a linear combination of weighted gene expression as below:
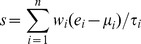
Here, *s* is the risk score of the patient; *n* is the number of differentially expressed genes; *w_i_* denotes the weight of gene *i*; *e_i_* denotes the expression level of gene *i*; and *μ_i_* and *τ_i_* are the mean and standard deviation of the gene expression values for gene *i* across all samples, respectively. Patients were then divided into high-score and low-score groups with the median of the risk score as the threshold value. A high score indicated a poor outcome. The weight of each gene was fixed, based on the training groups, and then tested in the validation groups [12]. Overall survival was analyzed by the Kaplan-Meier method. Differences in survival were tested for statistical significance by the log-rank test. *P*-values of less than 0.05 were considered to indicate statistical significance. The *survival* library of the R Statistical Package [19] was used to conduct survival analysis on the risk score.

## Results

### nmMLCK-mediated mouse genes

**Figure 1:**
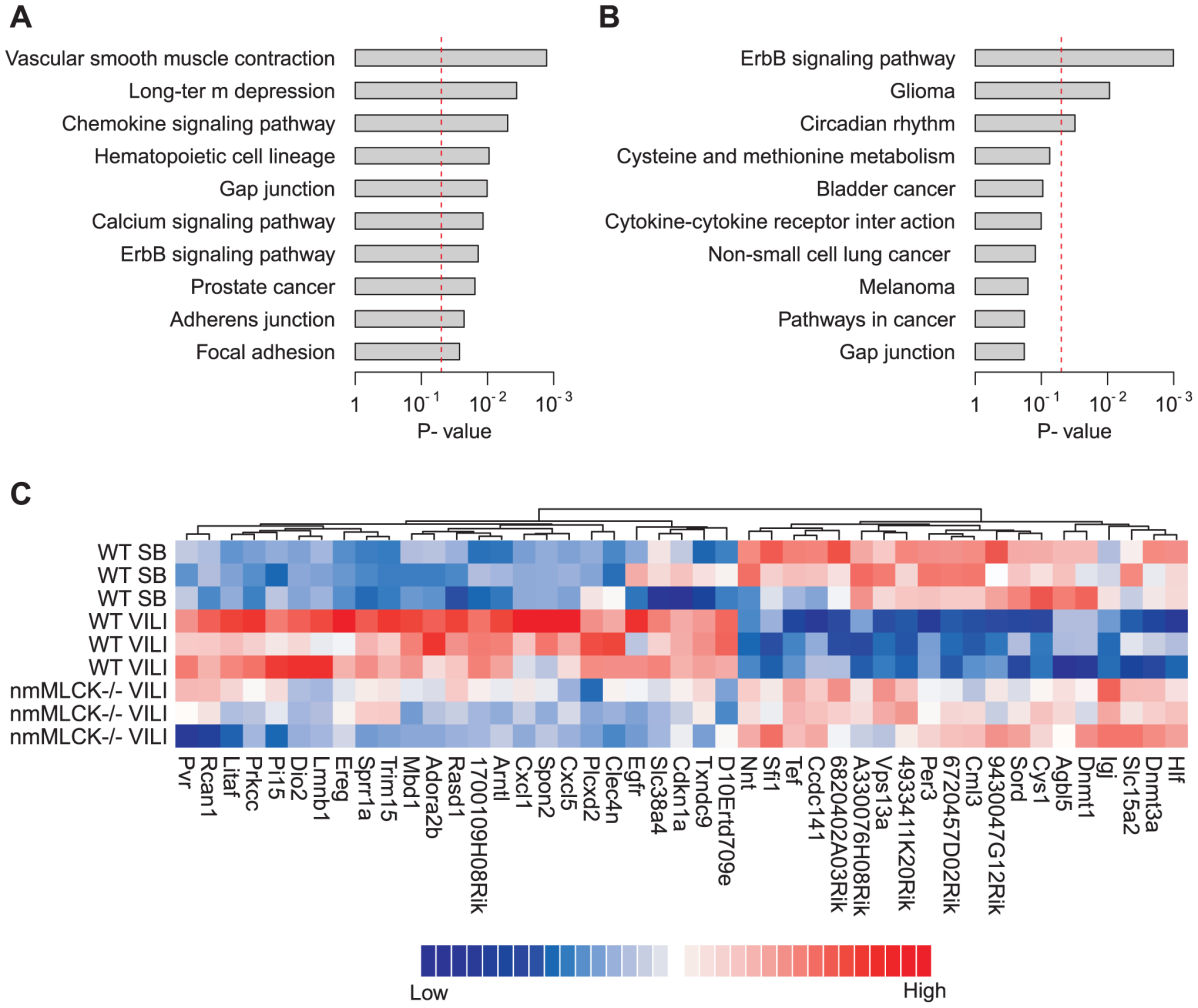
nmMLCK-mediated mouse genes. (A) Enriched pathways among the differentially expressed genes between WT VILI-exposed and VILI-exposed nmMLCK KO mice. The red line indicates the cutoff of significance (*P*<0.05). (B) Enriched pathways among the 45 nmMLCK-mediated genes. The red line indicates the cutoff of significance (*P*<0.05). (C) Heatmap of expression for WT control, WT VILI-exposed, and VILI-exposed nmMLCK KO mice. Red represents increased gene expression; Blue represents down-regulation.

At the specified significance level (fold-change >2 and FDR<10%), 365 genes were found be differentially expressed between VILI-exposed WT and nmMLCK KO mice, among which 117 genes were up-regulated while 248 genes were down-regulated in nmMLCK KO mice (Table S1). Several pathways were significantly enriched among these differentially expressed genes (*P*<0.05), such as vascular smooth muscle contraction, chemokine signaling pathway, calcium signaling pathway, ErbB signaling pathway, focal adhesion and so on (Figure 1A).

To filter out the top genes potentially associated with nmMLCK, we also compared the gene expression between WT control and VILI-exposed WT mice. 981 genes were found to be differentially expressed (fold-change >2 and FDR<10%) between these two groups (Table S2). Among these genes, we retained the genes with opposite direction of differential expression comparing Table S1 and S2. In other words, only the genes with attenuated VILI-mediated gene expression in nmMLCK KO mice were considered here. This step yielded 53 mouse genes. Lastly, we excluded the genes differentially expressed between WT control and VILI-exposed nmMLCK KO mice. In total, we retained 45 mouse genes for further study. Pathway analysis demonstrated a significant linkage of this gene set to ErbB signaling pathway, Glioma, Circadian rhythm (Figure 1B), which suggests that nmMLCK signaling contributes to the development and malignancy of tumors. Expression heatmap indicates that the expression profile of the 45 mouse genes were recovered at approximately normal levels of expression in nmMLCK KO mice exposed to VILI (Figure 1C). We deemed these 45 mouse genes as nmMLCK-mediated gene set (Table 1).

**Table 1:** nmMLCK-mediated mouse genes.

		WT control vs. WT VILI		WT VILI vs. KO VILI	
Gene symbol	Gene title	FC^a^	FDR	FC^b^	FDR
*Litaf*	LPS-induced TN factor	2.48	<0.001	0.47	<0.001
*Cxcl5*	chemokine (C-X-C motif) ligand 5	5.44	<0.001	0.22	<0.001
*Prkcc*	protein kinase C, gamma	4.03	<0.001	0.37	<0.001
*Cdkn1a*	cyclin-dependent kinase inhibitor 1A (P21)	4.02	<0.001	0.44	<0.001
*Lmnb1*	lamin B1	2.14	<0.001	0.49	<0.001
*Rasd1*	RAS, dexamethasone-induced 1	3.53	<0.001	0.48	<0.001
*Arntl*	aryl hydrocarbon receptor nuclear translocator-like	9.95	<0.001	0.18	<0.001
*Clec4n*	C-type lectin domain family 4, member n	2.45	<0.001	0.45	<0.001
*Dio2*	deiodinase, iodothyronine, type II	5.57	<0.001	0.21	<0.001
*Plcxd2*	phosphatidylinositol-specific phospholipase C, X domain containing 2	2.78	<0.001	0.26	<0.001
*Txndc9*	thioredoxin domain containing 9	4.63	<0.001	0.42	<0.001
*Slc38a4*	solute carrier family 38, member 4	2.24	<0.001	0.47	<0.001
*Sprr1a*	small proline-rich protein 1A	4.51	<0.001	0.45	<0.001
*Adora2b*	adenosine A2b receptor	4.71	<0.001	0.28	<0.001
*1700109H08Rik*	RIKEN cDNA 1700109H08 gene	3.51	<0.001	0.40	<0.001
*Mbd1*	methyl-CpG binding domain protein 1	2.37	<0.001	0.47	<0.001
*D10Ertd709e*	DNA segment, Chr 10, ERATO Doi 709, expressed	2.18	<0.001	0.48	<0.001
*Rcan1*	regulator of calcineurin 1	2.89	<0.001	0.44	0.051
*Spon2*	spondin 2, extracellular matrix protein	3.51	<0.001	0.35	0.051
*Ereg*	epiregulin	4.19	<0.001	0.44	0.051
*Pi15*	peptidase inhibitor 15	3.48	<0.001	0.38	0.051
*Pvr*	poliovirus receptor	2.90	<0.001	0.38	0.051
*Egfr*	epidermal growth factor receptor	2.32	0.052	0.38	0.051
*Trim15*	tripartite motif-containing 15	6.31	<0.001	0.41	0.051
*Cxcl1*	chemokine (C-X-C motif) ligand 1	2.62	<0.001	0.49	0.051
*Nnt*	nicotinamide nucleotide transhydrogenase	0.47	<0.001	2.18	<0.001
*Slc15a2*	solute carrier family 15 (H+/peptide transporter), member 2	0.38	0.029	3.59	<0.001
*Cys1*	cystin 1	0.29	<0.001	2.52	<0.001
*4933411K20Rik*	RIKEN cDNA 4933411K20 gene	0.45	<0.001	2.10	<0.001
*Dnmt3a*	DNA methyltransferase 3A	0.31	<0.001	4.15	<0.001
*Igj*	immunoglobulin joining chain	0.43	<0.001	8.34	<0.001
*Cml3*	camello-like 3	0.27	<0.001	2.33	<0.001
*6820402A03Rik*	RIKEN cDNA 6820402A03 gene	0.47	<0.001	2.04	<0.001
*Hlf*	hepatic leukemia factor	0.30	<0.001	3.34	<0.001
*Tef*	thyrotroph embryonic factor	0.43	<0.001	2.78	<0.001
*Sord*	sorbitol dehydrogenase	0.34	<0.001	2.18	<0.001
*Agbl5*	ATP/GTP binding protein-like 5	0.34	<0.001	2.09	<0.001
*Vps13a*	vacuolar protein sorting 13A (yeast)	0.43	<0.001	2.11	<0.001
*2610301F02Rik*	RIKEN cDNA 2610301F02 gene	0.41	0.075	3.03	<0.001
*Dnmt1*	DNA methyltransferase (cytosine-5) 1	0.32	<0.001	2.46	<0.001
*Sfi1*	Sfi1 homolog, spindle assembly associated (yeast)	0.42	<0.001	2.24	<0.001
*A330076H08Rik*	RIKEN cDNA A330076H08 gene	0.27	<0.001	2.06	<0.001
*6720457D02Rik*	RIKEN cDNA 6720457D02 gene	0.38	<0.001	2.01	<0.001
*Per3*	period homolog 3 (Drosophila)	0.19	<0.001	2.75	<0.001
*9430047G12Rik*	RIKEN cDNA 9430047G12 gene	0.38	<0.001	2.06	<0.001

a FC: fold change, which is calculated by dividing the expression in VILI-exposed WT mice by the expression in WT control mice.

b FC: fold change, which is calculated by dividing the expression in VILI-exposed nmMLCK KO mice by the expression in WT VILI-exposed mice.

### Prognostic molecular signature

nmMLCK is potentially involved in the pathogenesis of cancers [3], [4], [26]. To determine whether nmMLCK-mediated genes derived from nmMLCK KO mouse model were relevant to human cancers, we matched the 45 nmMLCK-mediated mouse genes to 38 distinct human orthologs (M38 signature) according to the definition of GeneCards [27], [28] (Table 2). We hypothesized that the M38 signature would be predictive of tumor outcome in cancer patients.

**Table 2:** M38 signature.

Mouse	Human	Gene title
*Adora2b*	*ADORA2B*	adenosine A2b receptor
*Agbl5*	*AGBL5*	ATP/GTP binding protein-like 5
*Arntl*	*ARNTL*	aryl hydrocarbon receptor nuclear translocator-like
*Ccdc141*	*CCDC141*	coiled-coil domain containing 141
*Cdkn1a*	*CDKN1A*	cyclin-dependent kinase inhibitor 1A (p21, Cip1)
*Clec4n*	*CLEC6A*	C-type lectin domain family 6, member A
*Cxcl1*	*CXCL2*	chemokine (C-X-C motif) ligand 2
*Cxcl5*	*CXCL6*	chemokine (C-X-C motif) ligand 6 (granulocyte chemotactic protein 2)
*Cys1*	*CYS1*	cystin 1
*Dio2*	*DIO2*	deiodinase, iodothyronine, type II
*Dnmt1*	*DNMT1*	DNA (cytosine-5-)-methyltransferase 1
*Dnmt3a*	*DNMT3A*	DNA (cytosine-5-)-methyltransferase 3 alpha
*Egfr*	*EGFR*	epidermal growth factor receptor
*Ereg*	*EREG*	epiregulin
*Hlf*	*HLF*	hepatic leukemia factor
*Igj*	*IGJ*	immunoglobulin J polypeptide, linker protein for immunoglobulin alpha and mu polypeptides
*4933411K20Rik*	*KIAA1430*	KIAA1430
*Litaf*	*LITAF*	lipopolysaccharide-induced TNF factor
*Lmnb1*	*LMNB1*	lamin B1
*Mbd1*	*MBD1*	methyl-CpG binding domain protein 1
*Nnt*	*NNT*	nicotinamide nucleotide transhydrogenase
*Per3*	*PER3*	period homolog 3 (Drosophila)
*Pi15*	*PI15*	peptidase inhibitor 15
*Plcxd2*	*PLCXD2*	phosphatidylinositol-specific phospholipase C, X domain containing 2
*Prkcc*	*PRKCG*	protein kinase C, gamma
*Pvr*	*PVR*	poliovirus receptor
*Rasd1*	*RASD1*	RAS, dexamethasone-induced 1
*Rcan1*	*RCAN1*	regulator of calcineurin 1
*Sfi1*	*SFI1*	Sfi1 homolog, spindle assembly associated (yeast)
*Slc15a2*	*SLC15A2*	solute carrier family 15 (H+/peptide transporter), member 2
*Slc38a4*	*SLC38A4*	solute carrier family 38, member 4
*Sord*	*SORD*	sorbitol dehydrogenase
*Spon2*	*SPON2*	spondin 2, extracellular matrix protein
*Sprr1a*	*SPRR1A*	small proline-rich protein 1A
*Tef*	*TEF*	thyrotrophic embryonic factor
*Trim15*	*TRIM15*	tripartite motif containing 15
*Txndc9*	*TXNDC9*	thioredoxin domain containing 9
*Vps13a*	*VPS13A*	vacuolar protein sorting 13 homolog A (S. cerevisiae)

**Figure 2:**
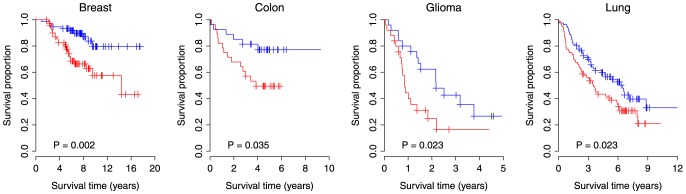
Expression of M38 signature predicts poor clinical outcome in multiple human cancers. Kaplan-Meier survival curves for patient groups identified by M38 risk score. Red curves are for the high-score patients while blue curves are for the low-score patients. High-score patients are defined as those having a M38 risk score greater than or equal to the group median score. *P*-values indicate significant differences in overall survival as measured by log-rank tests.

We constructed a risk scoring system that combined gene expression of M38 with risk for death in the training dataset. High-score patients were defined as those having a risk score greater than or equal to the group median score. In independent validation cohorts, we tested the ability of the risk score to stratify patients into prognostic groups. We performed Kaplan-Meier survival analysis comparing the high-score and low-score groups and determined statistical significance by log-rank tests. As expected, the M38 signature was able to identify patients with poor overall survival in breast cancer (*P*<0.001), colon cancer (*P*<0.001), glioma (*P*<0.001), and lung cancer (*P*<0.001) in the training cohorts (Figure S1). In the validation cohorts, Kaplan-Meier survival analysis comparing patient groups demonstrated a significantly reduced overall survival for high-score patients of breast cancer (*P* = 0.002), colon cancer (*P* = 0.035), glioma (*P* = 0.023), and lung cancer (*P* = 0.023) (Figure 2). The association between M38 risk score and overall survival was also confirmed by univariate Cox proportional hazard analysis of overall survival in both training and validation cohorts (Table 3). In the validation cohorts, high-score patients had an increased risk for death of 3.10-fold in breast cancer, 2.96-fold in colon cancer, 2.23-fold in glioma, and 1.60-fold in lung cancer.

**Table 3:** Univariate Cox proportional hazards regression of overall survival against M38 signature status.

	Training			Testing		
Cancer	Hazard ratio	95% Confidence interval	*P*-value	Hazard ratio	95% Confidence interval	*P*-value
Breast	4.50	(2.22, 9.12)	<0.001	3.10	(1.48, 6.49)	0.003
Colon	2.76	(1.69, 4.53)	<0.001	2.96	(1.13, 7.71)	0.027
Glioma	2.74	(1.63, 4.63)	<0.001	2.23	(1.10, 4.52)	0.026
Lung	2.55	(1.70, 3.84)	<0.001	1.60	(1.06, 2.41)	0.024

### Independence of M38 from other clinicopathologic factors

We investigated the performance of the M38 signature in comparison with clinicopathologic variables associated with prognosis in human cancers. A multivariate Cox analysis of overall survival indicated that M38 status remained a significant covariate in relation to the standard clinicopathologic factors in the four types of human cancers, such as patient age, lymph node status, tumor size, tumor grade, and so on (Table 4). We next stratified the patients according to the factors significant on multivariate analysis.

**Table 4:** Multivariate Cox proportional hazards regression of overall survival.

	Covariate	Hazard ratio	95% Confidence interval	*P*-value
Breast cancer	M38 high-score vs. low-score	1.47	(1.13, 1.92)	0.005
	Age (per year)	0.95	(0.91, 0.99)	0.016
	Lymph node status (+) vs. (−)	1.04	(0.66, 1.64)	0.878
	Tumor size ≥T2 vs. <T2	1.58	(0.99, 2.52)	0.054
	Tumor grade 2,3 vs. 1	4.26	(1.51, 12.01)	0. 006
	ER (−) vs. (+)	1.93	(1.20, 3.11)	0.007
				
Colon cancer	M38 high-score vs. low-score	1.55	(1.23, 1.94)	<0.001
	Age (per year)	1.03	(1.01, 1.05)	0.001
	Tumor grade 2,3 vs. 1	1.12	(0.40, 3.10)	0.828
	Stage	2.68	(2.01, 3.56)	<0.001
				
Glioma	M38 high-score vs. low-score	1.46	(1.11, 1.92)	0.006
	Age (per year)	1.02	(1.00, 1.05)	0.029
				
Lung cancer	M38 high-score vs. low-score	1.35	(1.16, 1.57)	<0.001
	Age (per year)	1.04	(1.02, 1.06)	<0.001
	Lymph node status (+) vs. (−)	2.49	(1.85, 3.34)	<0.001
	Tumor size ≥T3 vs. <T3	2.46	(1.66, 3.65)	<0.001
	Tumor grade 2,3 vs. 1	0.81	(0.52, 1.27)	0.359

For breast cancer, patients were stratified by age, tumor grade, and estrogen receptor (ER) status, respectively. For patients with age <40 and ≥40, the high-score ones had a significantly increased risk for death of 6.36-fold (*P*<0.001) and 2.80-fold (*P* = 0.001), respectively. For patients with tumor grade <2 and ≥2, the high-score patients had an increased risk for death of 2.63-fold (*P* = 0.410) and 2.65-fold (*P*<0.001), respectively. For patients with negative and positive ER status, the high-score patients had a significantly increased risk for death of 2.25-fold (*P* = 0.025) and 4.00-fold (*P*<0.001), respectively.

For colon cancer, patients were grouped by age and clinical stage, respectively. For patients with age <60 and ≥60, the high-score ones had a significantly increased risk for death of 2.29-fold (*P* = 0.025) and 2.88-fold (*P*<0.001), respectively. For patients with stage <3and ≥3, the high-score ones had a significantly increased risk for death of 3.50-fold (*P* = 0.015) and 1.71-fold (*P* = 0.024), respectively.

Patients with glioma were grouped by age. For patients with age <45 and ≥45, the high-score ones had a significantly increased risk for death of 3.46-fold (*P* = 0.004) and 2.00-fold (*P* = 0.045), respectively.

Lung cancer patients were stratified by age, lymph node status, and tumor size, respectively. For patients with age <65 and ≥65, the high-score ones had a significantly increased risk for death of 2.35-fold (*P*<0.001) and 1.97-fold (*P*<0.001), respectively. For patients with and without lymph node involvement, the high-score patients had a significantly increased risk for death of 1.62-fold (*P* = 0.012) and 1.73-fold (*P* = 0.014), respectively. For patients with tumor size <T3 and ≥T3, the high-score patients had an increased risk for death of 2.20-fold (*P*<0.001) and 1.63-fold (*P* = 0.180), respectively.

Kaplan-Meier survival analysis also demonstrated a significantly reduced overall survival for high-score patients in each subset grouped by each clinicopathologic factor (Figure S2-S5). Taken together, these results suggest that the expression of M38 signature is associated with clinical outcomes and is an independent prognostic factor.

## Discussion

This current study confirms an internal link between nmMLCK-mediate signaling and clinical cancer mortality with novel evidence: first, we defined a group of nmMLCK-driven genes with a murine model of lung inflammatory injury under which the effects of nmMLCK are amplified. Second. This nmMLCK-centralized molecular signature reflective of lung inflammatory gene expression is highly predictive of poor clinical outcome in four types of human cancer.

MLCK (gene code: *MYLK*) is a Ca^2+^/calmodulin-dependent kinase that phosphorylates myosin light chains (MLCs) to promote myosin interaction with cytoskeletal actin filaments [29]. It plays a key role in cytoskeleton rearrangement and contractile activities of both non-muscle tissue [30] and smooth muscle tissues [31]. The non-muscle isoform, nmMLCK, has been demonstrated to be a key participant in the inflammatory response based its ability to regulate vascular endothelium integrity and leukocyte influx from circulation into the lung broncoalveolar space [9]. Similar to pathogenesis in endothelial cells in ALI, cancer cell proliferation and migration require rapid dynamic regulation of the cytoskeleton, which is controlled by a group of cytoskeleton regulatory proteins, in which nmMLCK serves as a critical and central participant [1], [2]. In addition, trans-cellular extravasation, the essential step for malignant tumor metastasis, is well controlled by the activity of MLCK [3], [4]. Although still underestimated, MLCK started to be considered as a novel functional protein in cancer pathogenesis (initiation, proliferation, migration, and metastasis) [5], [6], [7]. This is especially true with the more widely expressed non-muscle isoform (nmMLCK).

Although little is known regarding the mechanisms of nmMLCK in the pathogenesis of tumor and its influence on the prognosis of human cancers, inflammatory response that regulated by nmMLCK in lungs is playing an active role in tumorogenesis and many successful therapies targeting chronic inflammation directly alter endothelial gene expression [32]. Murine VILI model amplifies the nmMLCK-mediated gene expression and serves as a satisfactory platform to dissect nmMLCK molecular signature in lung inflammatory injury.

Compared to a previous study [12], we used a non-conventional inflammation marker nmMLCK (compared to TNFα), which is more related to endothelial inflammation, as nmMLCK is selective expressed in non-muscle tissues such as endothelium[29]. Combined together, these two studies further verify the key role of “endothelial-specific” inflammation in cancer progression. Since nmMLCK is also expressed in other tissue types including epithelium and inflammatory leukocytes (same as TNFα), amplified molecular signature of nmMLCK by lung inflammation (M38 signature) might also involve other type of tissues in lungs, i.e., epithelium and infiltrated neutrophils. The potential contribution of M38 signature in pathogenesis in these tissues to cancer prognosis might also be important.

Our next study will focus on validation of these candidate genes filtered out in both nmMLCK and TNFα studies and generate a more accurate cancer prognosis platform with a refined gene set, which will lead to the development of cancer risk prediction/prognosis gene array in clinical trials.

*MYLK* is not in the M38 gene list, although the 38 genes were based on nmMLCK knockout mice. The possible complex reason might be that nmMLCK (210 Kd) is an isotype of *MYLK* gene product, while *MYLK* also produces smMLCK (108 Kd), which comprises of >80% of the *MYLK* gene products in lung. nmMLCK knockout does not interfere with smMLCK expression, but the microarray platform does not differentiate nmMLCK from smMLCK. This fact drives successful filtration of the 38 nmMLCK-mediated genes, but *MYLK* was not able to survive in the M38 gene list. To address the effect of *MYLK* in cancer survival prediction, we re-analyzed our datasets with the 39 genes (M38 genes plus *MYLK*), but no obvious improvement was found (Table S3). Nevertheless, several recent studies indicate that nmMLCK expression is indeed changed in human cancers, such as colorectal cancer [33] and prostate cancer [34].

We used a scoring system to assign a M38-based risk score to each patients. This scoring system can also be directly applied to other published cancer gene signatures. The comparison between cancer gene signatures can be simply conducted by comparing the prognostic power of the risk scores of individual gene signatures. In this study, we used the median of M38 score to divide the patents into two parts (high-score and low-score patients) to do categorized analyses (such as Kaplan-Meier analysis and log-rank test). Clinically, we can use zero as an absolute cutoff to stratify the patients into high-risk and low-risk groups, because the median of M38 score is approximately equal to zero in each dataset.

This study provides the first prognostic cancer gene signature derived from a murine model of nmMLCK-associated lung inflammation. Activation of nmMLCK-involved pathways contributes to tumor growth and progression in human cancers. These findings support the notion that nmMLCK is an attractive candidate molecular target in lung diseases.

## Supporting Information

Figure S1 **Application of the M38 signature to training datasets representing four human cancers.** Kaplan-Meier survival curves for patient groups identified by M38 risk score. Red curves are for the high-score patients while blue curves are for the low-score patients. High-score patients are defined as those having a M38 risk score greater than or equal to the group median score. *P*-values indicate significant differences in overall survival as measured by log-rank tests.
(PDF)

Figure S2 **M38 signature adds prognostic value to clinicopathologic factors associated with survival in human breast cancer.** Kaplan-Meier survival curves of patient cohorts grouped by (A) age, (B) tumor grade, or (C) ER status. Red curves are for the high-score patients while blue curves are for the low-score patients. High-score patients are defined as those having a M38 risk score greater than or equal to the group median score. *P*-values indicate significant differences in overall survival as measured by log-rank tests.
(PDF)

Figure S3 **M38 signature adds prognostic value to clinicopathologic factors associated with survival in human colon cancer.** Kaplan-Meier survival curves of patient cohorts grouped by (A) age or (B) clinical stage. Red curves are for the high-score patients while blue curves are for the low-score patients. High-score patients are defined as those having a M38 risk score greater than or equal to the group median score. *P*-values indicate significant differences in overall survival as measured by log-rank tests.
(PDF)

Figure S4 **M38 signature adds prognostic value to clinicopathologic factors associated with survival in human glioma.** Kaplan-Meier survival curves of patient cohorts grouped by age. Red curves are for the high-score patients while blue curves are for the low-score patients. High-score patients are defined as those having a M38 risk score greater than or equal to the group median score. *P*-values indicate significant differences in overall survival as measured by log-rank tests.
(PDF)

Figure S5 **M38 signature adds prognostic value to clinicopathologic factors associated with survival in human lung cancer.** Kaplan-Meier survival curves of patient cohorts grouped by (A) age, (B) lymph node status, or (C) tumor size. Red curves are for the high-score patients while blue curves are for the low-score patients. High-score patients are defined as those having a M38 risk score greater than or equal to the group median score. *P*-values indicate significant differences in overall survival as measured by log-rank tests.
(PDF)

Table S1 **Differentially expressed genes between VILI-exposed WT and VILI-exposed nmMLCK KO mice.**
(PDF)

Table S2 **Differentially expressed genes between WT control and VILI-exposed WT mice.**
(PDF)

Table S3 **Univariate Cox proportional hazards regression of overall survival against M38+MYLK signature status.**
(PDF)
