# Supplementary figures and images for: Genes Influenced by the Non-Muscle Isoform of Myosin Light Chain Kinase Impact Human Cancer Prognosis
Source: PLoS One. 2014 Apr 8;9(4):e94325. doi: 10.1371/journal.pone.0094325 (PMC3979809; doi:10.1371/journal.pone.0094325)

Breast

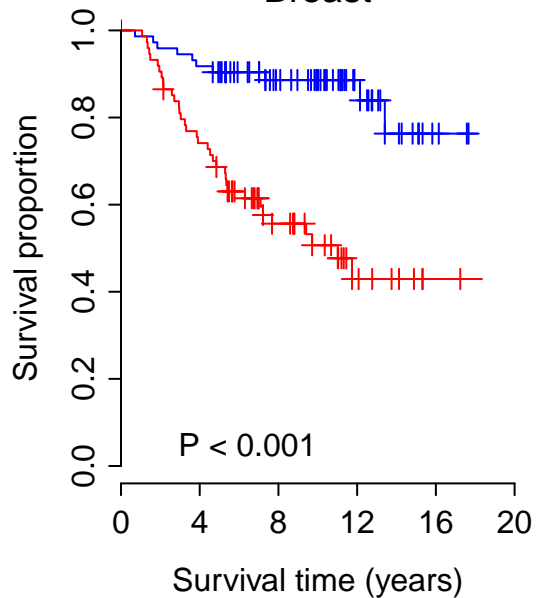

Colon

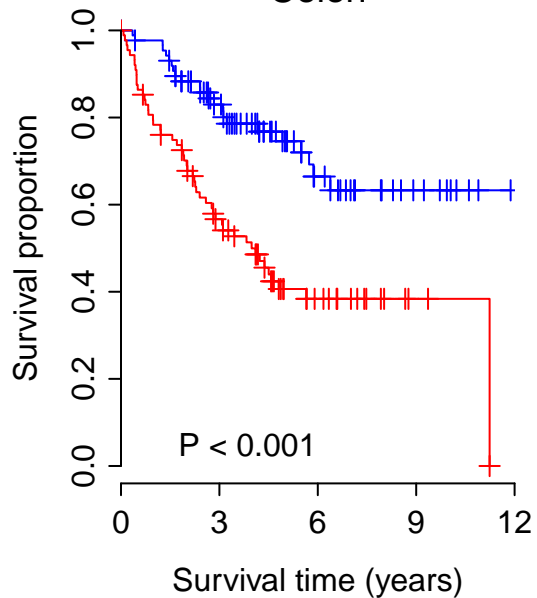

Glioma

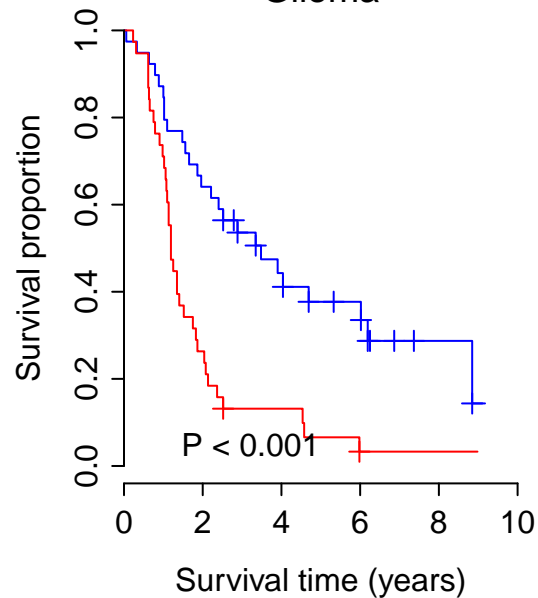

Lung

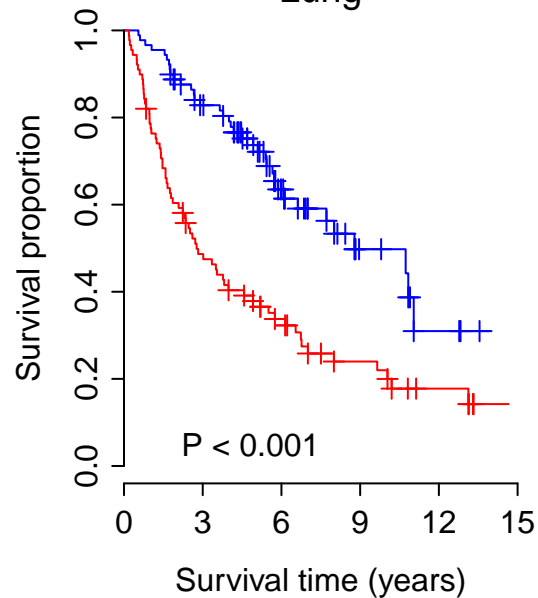

Supplement: Figure S1 — Application of the M38 signature to training datasets representing four human cancers. Kaplan-Meier survival curves for patient groups identified by M38 risk score. Red curves are for the high-score patients while blue curves are for the low-score patients. High-score patients are defined as those having a M38 risk score greater than or equal to the group median score. P-values indicate significant differences in overall survival as measured by log-rank tests. (PDF) [file pone.0094325.s001.pdf]

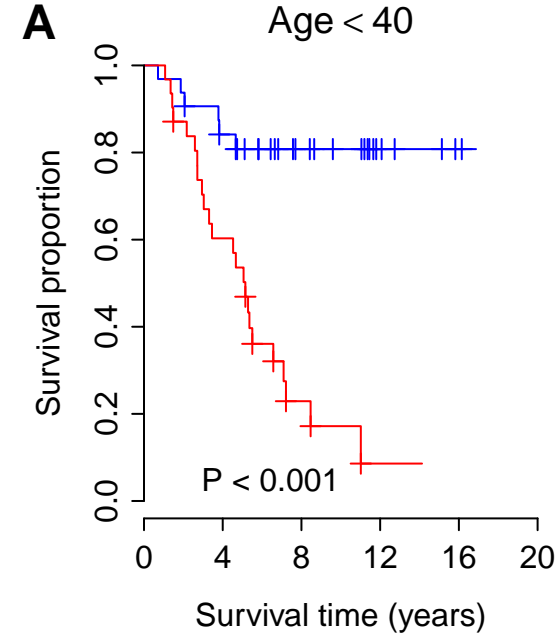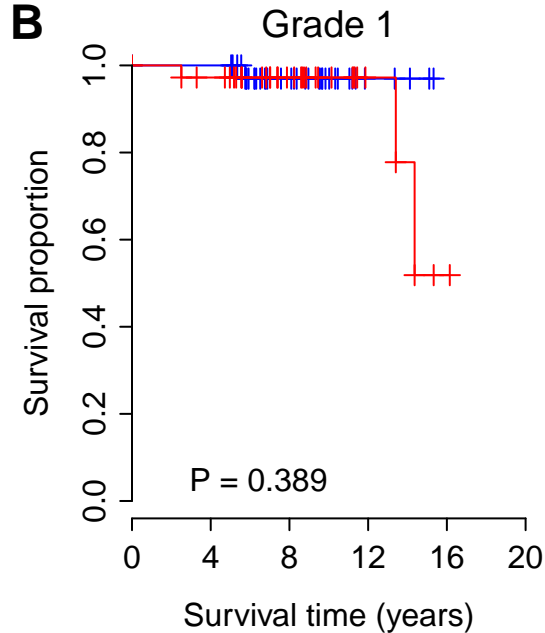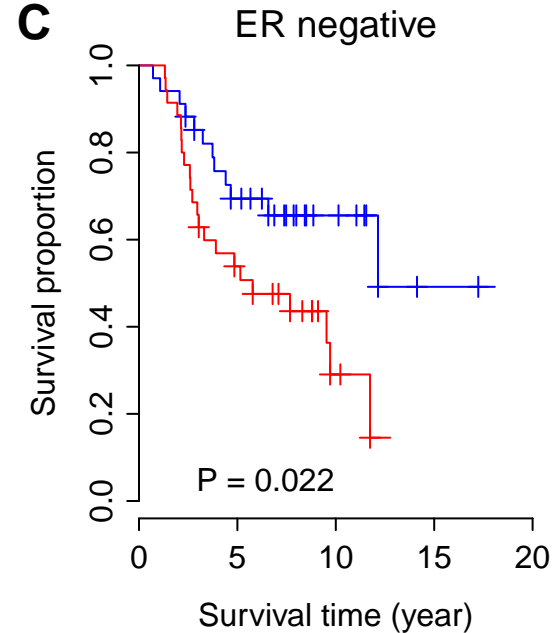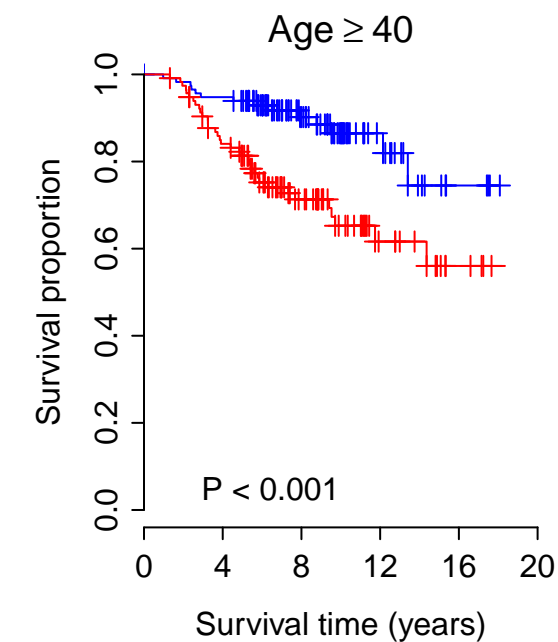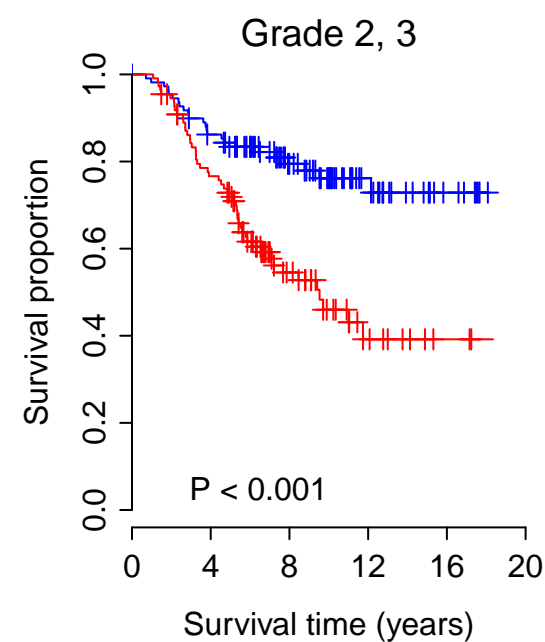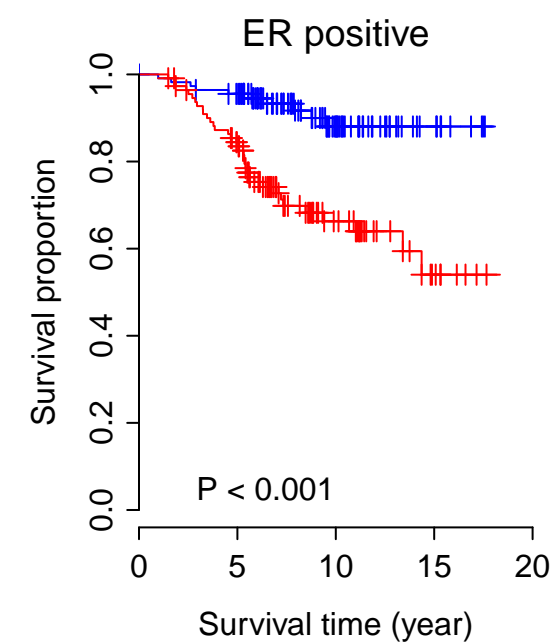

Supplement: Figure S2 — M38 signature adds prognostic value to clinicopathologic factors associated with survival in human breast cancer. Kaplan-Meier survival curves of patient cohorts grouped by (A) age, (B) tumor grade, or (C) ER status. Red curves are for the high-score patients while blue curves are for the low-score patients. High-score patients are defined as those having a M38 risk score greater than or equal to the group median score. P-values indicate significant differences in overall survival as measured by log-rank tests. (PDF) [file pone.0094325.s002.pdf]

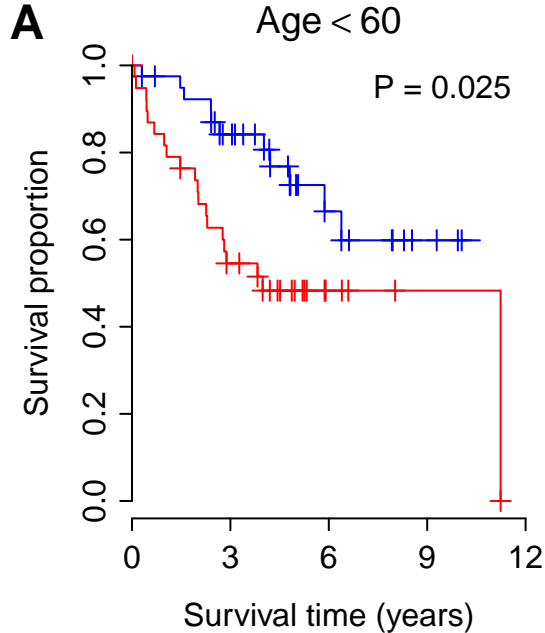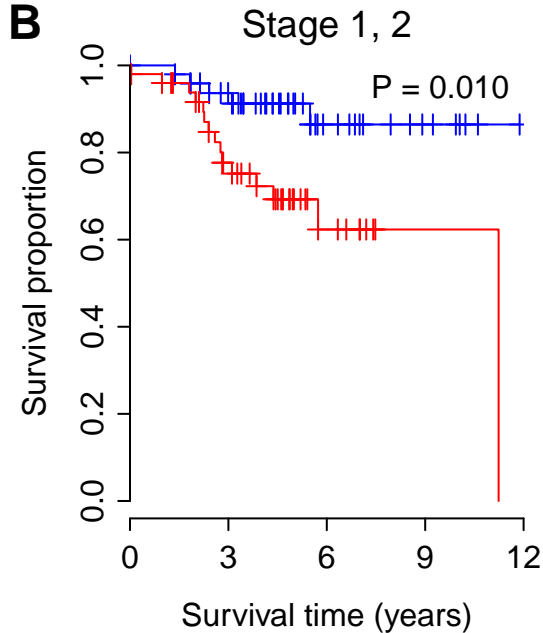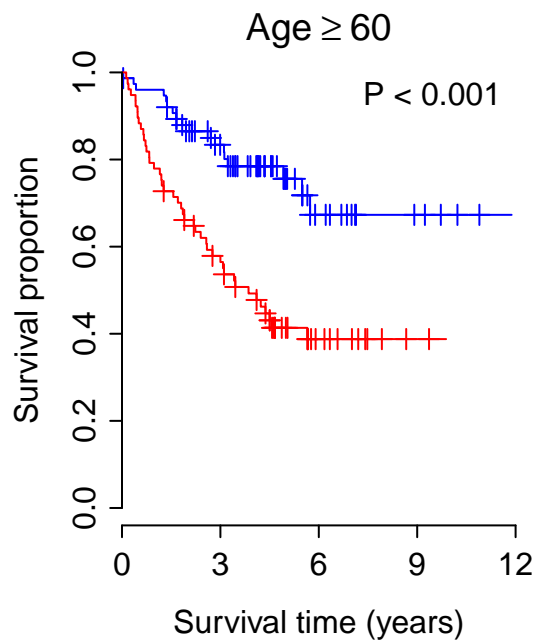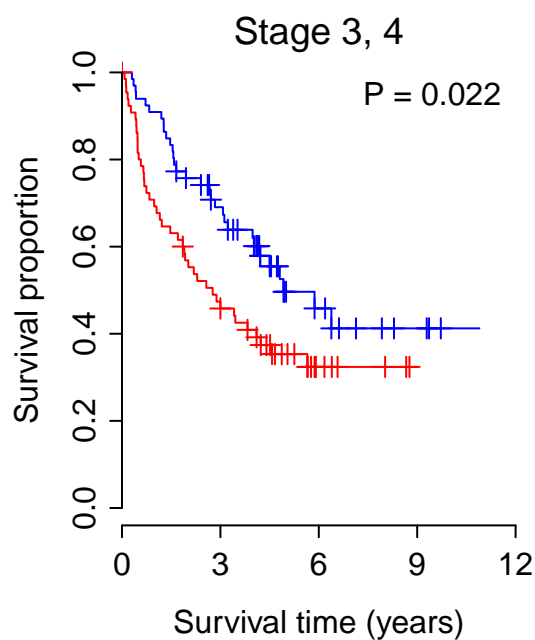

Supplement: Figure S3 — M38 signature adds prognostic value to clinicopathologic factors associated with survival in human colon cancer. Kaplan-Meier survival curves of patient cohorts grouped by (A) age or (B) clinical stage. Red curves are for the high-score patients while blue curves are for the low-score patients. High-score patients are defined as those having a M38 risk score greater than or equal to the group median score. P-values indicate significant differences in overall survival as measured by log-rank tests. (PDF) [file pone.0094325.s003.pdf]

Age < 45

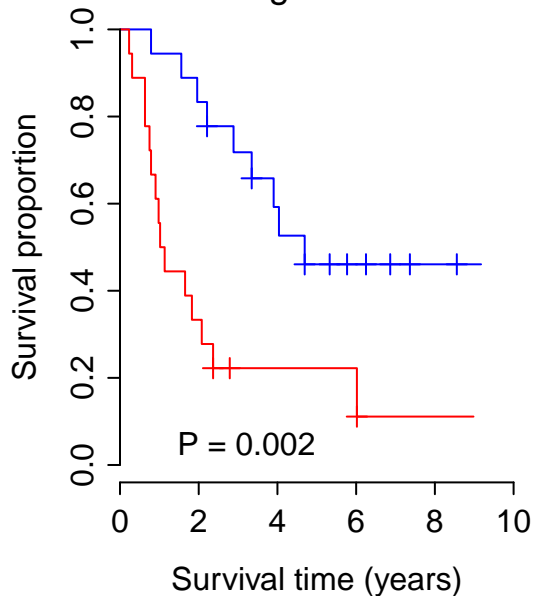

Age  $\geq 45$

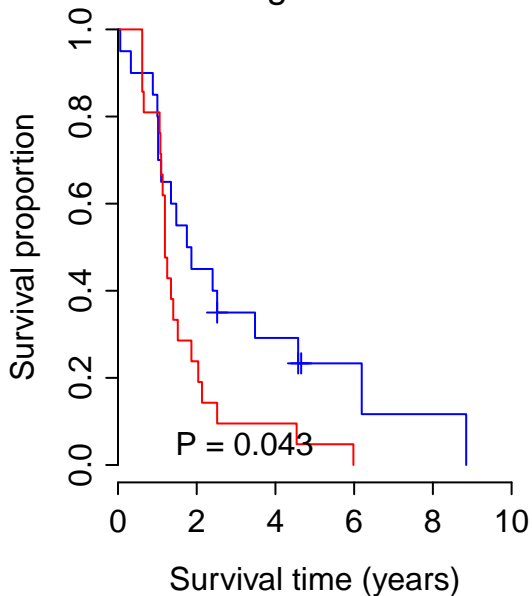

Supplement: Figure S4 — M38 signature adds prognostic value to clinicopathologic factors associated with survival in human glioma. Kaplan-Meier survival curves of patient cohorts grouped by age. Red curves are for the high-score patients while blue curves are for the low-score patients. High-score patients are defined as those having a M38 risk score greater than or equal to the group median score. P-values indicate significant differences in overall survival as measured by log-rank tests. (PDF) [file pone.0094325.s004.pdf]

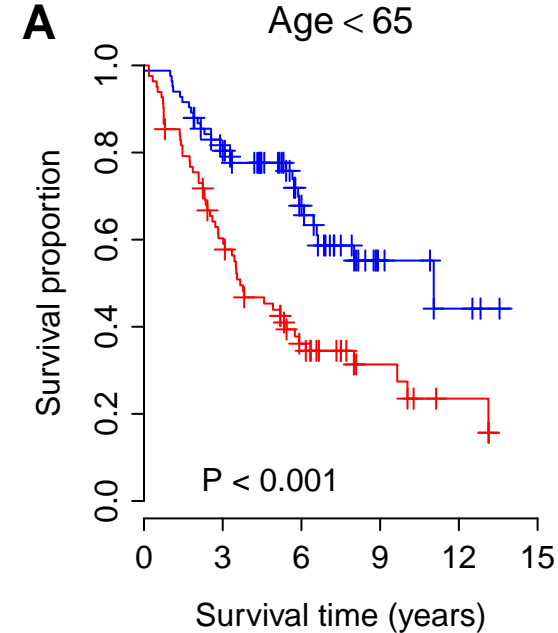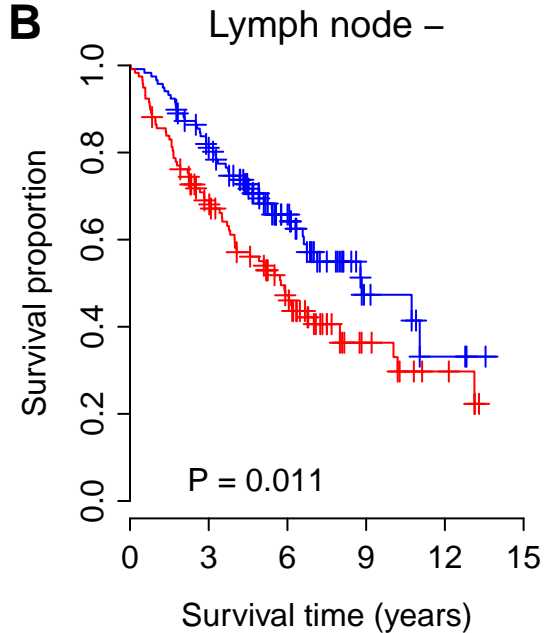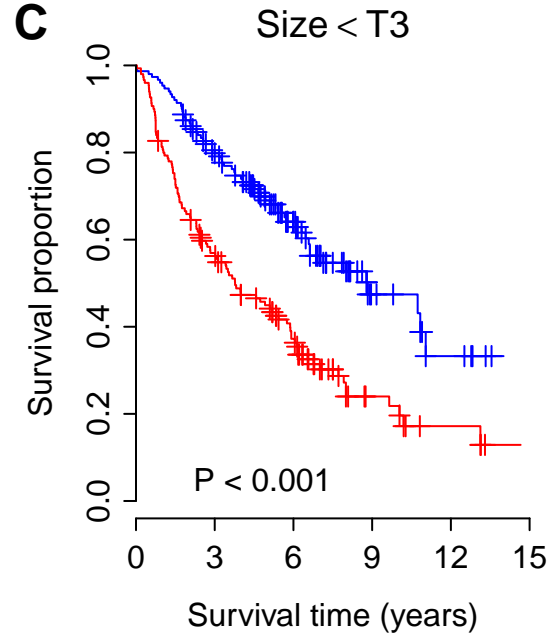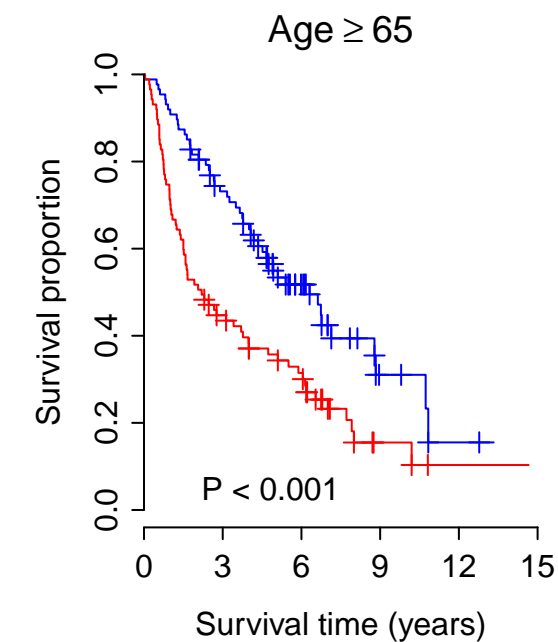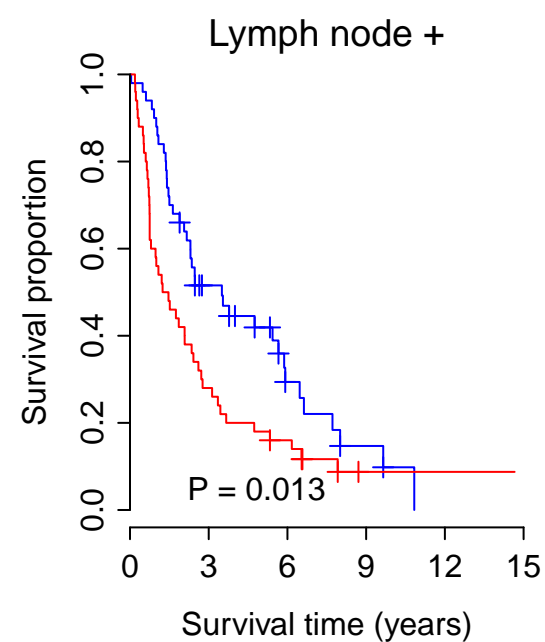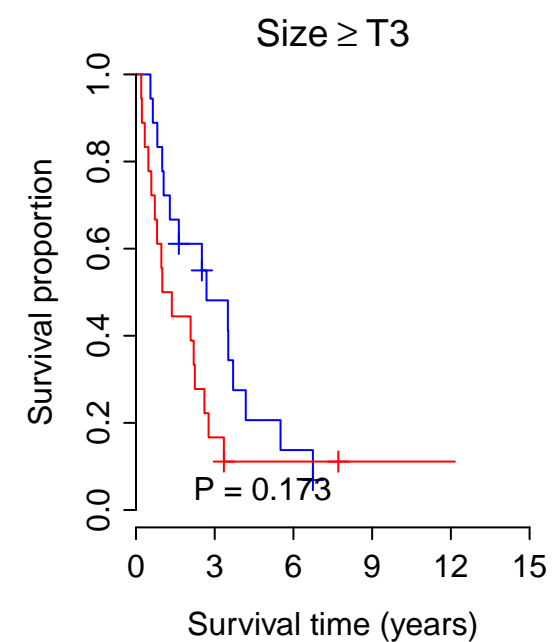

Supplement: Figure S5 — M38 signature adds prognostic value to clinicopathologic factors associated with survival in human lung cancer. Kaplan-Meier survival curves of patient cohorts grouped by (A) age, (B) lymph node status, or (C) tumor size. Red curves are for the high-score patients while blue curves are for the low-score patients. High-score patients are defined as those having a M38 risk score greater than or equal to the group median score. P-values indicate significant differences in overall survival as measured by log-rank tests. (PDF) [file pone.0094325.s005.pdf]
