# Supplementary material for: Genes Influenced by the Non-Muscle Isoform of Myosin Light Chain Kinase Impact Human Cancer Prognosis
Source: PLoS One. 2014 Apr 8;9(4):e94325. doi: 10.1371/journal.pone.0094325 (PMC3979809; doi:10.1371/journal.pone.0094325)
Supplement: Table S1 — Differentially expressed genes between VILI-exposed WT and VILI-exposed nmMLCK KO mice. (PDF) [file pone.0094325.s006.pdf]

Table S1. Differentially expressed genes between VILI-exposed WT and VILI-exposed nmMLCK KO mice

Gene symbol	Gene title	FC[a]	FDR
2900097C17Rik	RIKEN cDNA 2900097C17 gene	0.17	0.051
Arntl	aryl hydrocarbon receptor nuclear translocator-like	0.18	< 0.001
Ptplad1	protein tyrosine phosphatase-like A domain containing 1	0.19	0.051
Upk1b	uroplakin 1B	0.19	< 0.001
5430404G13Rik	RIKEN cDNA 5430404G13 gene	0.19	< 0.001
5330426P16Rik	RIKEN cDNA 5330426P16 gene	0.22	< 0.001
Cxcl5	chemokine (C-X-C motif) ligand 5	0.22	< 0.001
Rab27a	RAB27A, member RAS oncogene family	0.23	< 0.001
Gja1	gap junction protein, alpha 1	0.23	0.051
S1pr3	sphingosine-1-phosphate receptor 3	0.23	0.051
Cyth1	cytohesin 1	0.23	< 0.001
Il6st	interleukin 6 signal transducer	0.24	0.051
Cxcl2	chemokine (C-X-C motif) ligand 2	0.24	0.051
AI848100	expressed sequence AI848100	0.24	< 0.001
Mbtps1	membrane-bound transcription factor peptidase, site 1	0.24	0.051
C330007P06Rik	RIKEN cDNA C330007P06 gene	0.24	< 0.001
Stom	stomatin	0.24	< 0.001
Arl15	ADP-ribosylation factor-like 15	0.26	< 0.001
Fbxw11	F-box and WD-40 domain protein 11	0.26	0.051
Trim35	tripartite motif-containing 35	0.27	0.051
Epb4.1l2	erythrocyte protein band 4.1-like 2	0.27	0.051
Ptx3	pentraxin related gene	0.28	0.051
Ndst1	N-deacetylase/N-sulfotransferase (heparan glucosaminyl) 1	0.30	0.051
Calcrl	calcitonin receptor-like	0.30	0.051
Tmem33	transmembrane protein 33	0.30	< 0.001
Abca1	ATP-binding cassette, sub-family A (ABC1), member 1	0.30	0.051
Mme	membrane metallo endopeptidase	0.30	< 0.001
Tm9sf3	transmembrane 9 superfamily member 3	0.31	0.051
Jhdm1d	jumonji C domain-containing histone demethylase 1 homolog D (S. cerevisiae)	0.31	0.051
Spry4	sprouty homolog 4 (Drosophila)	0.31	0.051
Spty2d1	SPT2, Suppressor of Ty, domain containing 1 (S. cerevisiae)	0.31	< 0.001
Osmr	oncostatin M receptor	0.31	< 0.001
Ide	insulin degrading enzyme	0.31	< 0.001
Sigmar1	sigma non-opioid intracellular receptor 1	0.32	< 0.001
Adamts4	a disintegrin-like and metallopeptidase (reprolysin type) with thrombospondin type 1 motif, 4	0.32	< 0.001
Mtpn	myotrophin	0.32	0.051
Stk38l	serine/threonine kinase 38 like	0.32	0.051
4833439L19Rik	RIKEN cDNA 4833439L19 gene	0.33	0.051
Podxl	podocalyxin-like	0.33	0.051
Ranbp6	RAN binding protein 6	0.33	< 0.001
Cd38	CD38 antigen	0.33	0.051
Slc23a2	solute carrier family 23 (nucleobase transporters), member 2	0.34	0.051
4932438A13Rik	RIKEN cDNA 4932438A13 gene	0.34	0.051
Map3k7	mitogen-activated protein kinase kinase kinase 7	0.34	< 0.001

Dep1	diabetic embryopathy 1	0.34	0.051
Ccl9	chemokine (C-C motif) ligand 9	0.34	< 0.001
Pkia	protein kinase inhibitor, alpha	0.34	0.051
Ptprk	protein tyrosine phosphatase, receptor type, K	0.35	0.051
Spon2	spondin 2, extracellular matrix protein	0.35	0.051
Lims1	LIM and senescent cell antigen-like domains 1	0.35	0.051
A630005I04Rik	RIKEN cDNA A630005I04 gene	0.35	< 0.001
Brcc3	BRCA1/BRCA2-containing complex, subunit 3	0.35	0.051
0610010K06Rik	RIKEN cDNA 0610010K06 gene	0.36	0.051
Tgfbr2	transforming growth factor, beta receptor II	0.36	0.051
Nr3c1	nuclear receptor subfamily 3, group C, member 1	0.36	0.051
Tollip	toll interacting protein	0.36	0.051
Ube3a	ubiquitin protein ligase E3A	0.36	0.051
Vamp3	vesicle-associated membrane protein 3	0.36	0.051
Msrb3	methionine sulfoxide reductase B3	0.36	< 0.001
Prkcc	protein kinase C, gamma	0.37	< 0.001
Arpp19	cAMP-regulated phosphoprotein 19	0.37	< 0.001
Hipk3	homeodomain interacting protein kinase 3	0.37	0.051
Atrx	alpha thalassemia/mental retardation syndrome X-linked homolog (human)	0.37	0.051
Gcnt2	glucosaminyl (N-acetyl) transferase 2, I-branching enzyme	0.37	0.051
Pvr	poliovirus receptor	0.38	0.051
Tmed5	transmembrane emp24 protein transport domain containing 5	0.38	0.051
Egfr	epidermal growth factor receptor	0.38	0.051
Pla2g15	phospholipase A2, group XV	0.38	0.051
1110018J18Rik	RIKEN cDNA 1110018J18 gene	0.38	0.051
Fam63a	family with sequence similarity 63, member A	0.38	0.051
Sfrp1	secreted frizzled-related protein 1	0.38	< 0.001
Smg1	SMG1 homolog, phosphatidylinositol 3-kinase-related kinase (C. elegans)	0.38	0.051
Pi15	peptidase inhibitor 15	0.38	0.051
Scamp1	secretory carrier membrane protein 1	0.38	0.051
Tgfbr1	transforming growth factor, beta receptor I	0.38	0.051
Gorab	golgin, RAB6-interacting	0.39	< 0.001
Abhd10	abhydrolase domain containing 10	0.39	< 0.001
Cd59a	CD59a antigen	0.39	< 0.001
Mobkl1b	MOB1, Mps One Binder kinase activator-like 1B (yeast)	0.39	0.051
Ccdc91	coiled-coil domain containing 91	0.39	< 0.001
Ube2b	ubiquitin-conjugating enzyme E2B, RAD6 homology (S. cerevisiae)	0.39	< 0.001
Atl2	atlastin GTPase 2	0.39	< 0.001
Pdlim5	PDZ and LIM domain 5	0.39	0.051
Ccnd2	cyclin D2	0.40	0.051
Garnl1	GTPase activating RANGAP domain-like 1	0.40	0.051
Bcl2l11	BCL2-like 11 (apoptosis facilitator)	0.40	0.051
Slc38a10	solute carrier family 38, member 10	0.40	< 0.001
Rbm18	RNA binding motif protein 18	0.40	0.051
Prkar2a	protein kinase, cAMP dependent regulatory, type II alpha	0.40	0.051
B230219D22Rik	RIKEN cDNA B230219D22 gene	0.40	< 0.001
Plcxd2	phosphatidylinositol-specific phospholipase C, X domain	0.40	< 0.001

	containing 2		
Abcb7	ATP-binding cassette, sub-family B (MDR/TAP), member 7	0.40	< 0.001
Carhsp1	calcium regulated heat stable protein 1	0.40	< 0.001
Gna13	guanine nucleotide binding protein, alpha 13	0.40	< 0.001
1700109H08Rik	RIKEN cDNA 1700109H08 gene	0.40	< 0.001
1600029D21Rik	RIKEN cDNA 1600029D21 gene	0.40	< 0.001
Nktr	natural killer tumor recognition sequence	0.41	0.051
Tgoln1	trans-golgi network protein	0.41	0.051
Cybb	cytochrome b-245, beta polypeptide	0.41	< 0.001
Golph3	golgi phosphoprotein 3	0.41	0.051
Trim15	tripartite motif-containing 15	0.41	0.051
Prkab1	protein kinase, AMP-activated, beta 1 non-catalytic subunit	0.41	< 0.001
Lypla1	lysophospholipase 1	0.41	0.051
Zfand5	zinc finger, AN1-type domain 5	0.41	0.051
Plek	pleckstrin	0.41	0.051
Immt	inner membrane protein, mitochondrial	0.41	0.051
Cpeb4	cytoplasmic polyadenylation element binding protein 4	0.41	0.051
9030624J02Rik	RIKEN cDNA 9030624J02 gene	0.41	0.051
Alpl	alkaline phosphatase, liver/bone/kidney	0.41	< 0.001
Itm2a	integral membrane protein 2A	0.41	< 0.001
Ada	adenosine deaminase	0.42	< 0.001
2310035C23Rik	RIKEN cDNA 2310035C23 gene	0.42	0.051
Vtn	vitronectin	0.42	< 0.001
Rad52	RAD52 homolog (S. cerevisiae)	0.42	< 0.001
Txndc9	thioredoxin domain containing 9	0.42	< 0.001
Pdgfd	platelet-derived growth factor, D polypeptide	0.42	< 0.001
Pfkp	phosphofructokinase, platelet	0.42	0.051
Tmed7	transmembrane emp24 protein transport domain containing 7	0.42	0.051
Pfkfb3	6-phosphofructo-2-kinase/fructose-2,6-biphosphatase 3	0.42	0.051
Tnfsf9	tumor necrosis factor (ligand) superfamily, member 9	0.42	< 0.001
Vipr2	vasoactive intestinal peptide receptor 2	0.43	< 0.001
Tug1	taurine upregulated gene 1	0.43	0.051
Ahcyl1	S-adenosylhomocysteine hydrolase-like 1	0.43	0.051
Slc35a3	solute carrier family 35 (UDP-N-acetylglucosamine (UDP-GlcNAc) transporter), member 3	0.43	0.051
Ahcyl2	S-adenosylhomocysteine hydrolase-like 2	0.43	< 0.001
Arhgap29	Rho GTPase activating protein 29	0.43	0.051
Usp9x	ubiquitin specific peptidase 9, X chromosome	0.43	0.051
Pard6g	par-6 partitioning defective 6 homolog gamma (C. elegans)	0.43	< 0.001
Gna12	guanine nucleotide binding protein, alpha 12	0.43	0.051
Tfrc	transferrin receptor	0.43	0.051
Slc6a2	solute carrier family 6 (neurotransmitter transporter, noradrenalin), member 2	0.43	< 0.001
Twsg1	twisted gastrulation homolog 1 (Drosophila)	0.43	0.051
Ids	iduronate 2-sulfatase	0.43	0.051
Tifa	TRAF-interacting protein with forkhead-associated domain	0.44	0.051
Cyb5b	cytochrome b5 type B	0.44	0.051
Rcan1	regulator of calcineurin 1	0.44	0.051
Ereg	epiregulin	0.44	0.051
Mapk14	mitogen-activated protein kinase 14	0.44	0.051

Flt3l	FMS-like tyrosine kinase 3 ligand	0.44	< 0.001
Cdkn1a	cyclin-dependent kinase inhibitor 1A (P21)	0.44	< 0.001
Cugbp1	CUG triplet repeat, RNA binding protein 1	0.44	< 0.001
Klhl5	kelch-like 5 (Drosophila)	0.44	< 0.001
Adam17	a disintegrin and metallopeptidase domain 17	0.44	< 0.001
Has1	hyaluronan synthase1	0.44	0.051
C1galt1	core 1 synthase, glycoprotein-N-acetylgalactosamine 3-beta-galactosyltransferase, 1	0.44	0.051
Fam114a1	family with sequence similarity 114, member A1	0.44	< 0.001
1200011I18Rik	RIKEN cDNA 1200011I18 gene	0.45	0.051
Cpeb2	cytoplasmic polyadenylation element binding protein 2	0.45	0.051
Bdp1	B double prime 1, subunit of RNA polymerase III transcription initiation factor IIIB	0.45	< 0.001
Polr3c	polymerase (RNA) III (DNA directed) polypeptide C	0.45	0.051
Clec4n	C-type lectin domain family 4, member n	0.45	< 0.001
Nek6	NIMA (never in mitosis gene a)-related expressed kinase 6	0.45	< 0.001
Sprr1a	small proline-rich protein 1A	0.45	< 0.001
Rp2h	retinitis pigmentosa 2 homolog (human)	0.45	< 0.001
Plcb1	phospholipase C, beta 1	0.45	< 0.001
Stk3	serine/threonine kinase 3 (Ste20, yeast homolog)	0.45	0.051
Bicd2	bicaudal D homolog 2 (Drosophila)	0.46	< 0.001
Ptar1	protein prenyltransferase alpha subunit repeat containing 1	0.46	0.051
P4ha1	procollagen-proline, 2-oxoglutarate 4-dioxygenase (proline 4-hydroxylase), alpha 1 polypeptide	0.46	0.051
Vcam1	vascular cell adhesion molecule 1	0.46	< 0.001
Nol11	nucleolar protein 11	0.46	0.051
Ywhag	tyrosine 3-monooxygenase/tryptophan 5-monooxygenase activation protein, gamma polypeptide	0.46	0.051
Tspan2	tetraspanin 2	0.46	0.051
Nrp	neural regeneration protein	0.46	< 0.001
Rnf141	ring finger protein 141	0.46	< 0.001
Arid4b	AT rich interactive domain 4B (RBP1-like)	0.46	0.051
2210018M11Rik	RIKEN cDNA 2210018M11 gene	0.46	< 0.001
Vsnl1	visinin-like 1	0.46	< 0.001
Twf1	twinfilin, actin-binding protein, homolog 1 (Drosophila)	0.46	< 0.001
Entpd1	ectonucleoside triphosphate diphosphohydrolase 1	0.46	< 0.001
Slc35a5	solute carrier family 35, member A5	0.46	< 0.001
Tmed2	transmembrane emp24 domain trafficking protein 2	0.47	0.051
Litaf	LPS-induced TN factor	0.47	< 0.001
Mrpl50	mitochondrial ribosomal protein L50	0.47	< 0.001
Dcun1d1	DCN1, defective in cullin neddylation 1, domain containing 1 (S. cerevisiae)	0.47	0.051
Plekha3	pleckstrin homology domain-containing, family A (phosphoinositide binding specific) member 3	0.47	0.051
Mbd1	methyl-CpG binding domain protein 1	0.47	< 0.001
Hipk2	homeodomain interacting protein kinase 2	0.47	< 0.001
Slc38a4	solute carrier family 38, member 4	0.47	< 0.001
Elmo1	engulfment and cell motility 1, ced-12 homolog (C. elegans)	0.47	0.051
Dr1	down-regulator of transcription 1	0.47	0.051
Ap3m1	adaptor-related protein complex 3, mu 1 subunit	0.47	0.051

2700078E11Rik	RIKEN cDNA 2700078E11 gene	0.47	< 0.001
Kpna1	karyopherin (importin) alpha 1	0.47	< 0.001
Ubr1	ubiquitin protein ligase E3 component n-recognin 1	0.47	0.051
Wwc2	WW, C2 and coiled-coil domain containing 2	0.47	0.051
EG665955	predicted gene, EG665955	0.47	0.051
Nt5c2	5'-nucleotidase, cytosolic II	0.47	0.051
Gng2	guanine nucleotide binding protein (G protein), gamma 2	0.47	< 0.001
1500003O03Rik	RIKEN cDNA 1500003O03 gene	0.47	0.051
Gulp1	GULP, engulfment adaptor PTB domain containing 1	0.47	< 0.001
Rapgef1	Rap guanine nucleotide exchange factor (GEF) 1	0.47	< 0.001
Cacna2d1	calcium channel, voltage-dependent, alpha2/delta subunit 1	0.47	< 0.001
Cd44	CD44 antigen	0.47	0.051
Slc26a4	solute carrier family 26, member 4	0.47	< 0.001
Gosr1	golgi SNAP receptor complex member 1	0.48	0.051
Ppargc1a	peroxisome proliferative activated receptor, gamma, coactivator 1 alpha	0.48	< 0.001
Fam107a	family with sequence similarity 107, member A	0.48	< 0.001
Sel1l	sel-1 suppressor of lin-12-like (C. elegans)	0.48	0.051
Pdgfra	platelet derived growth factor receptor, alpha polypeptide	0.48	0.051
Gucy1a3	guanylate cyclase 1, soluble, alpha 3	0.48	0.051
Ddhd1	DDHD domain containing 1	0.48	< 0.001
Akt3	thymoma viral proto-oncogene 3	0.48	0.051
Pxn	paxillin	0.48	0.051
Rasd1	RAS, dexamethasone-induced 1	0.48	< 0.001
Hmgcs2	3-hydroxy-3-methylglutaryl-Coenzyme A synthase 2	0.48	< 0.001
Acsl4	acyl-CoA synthetase long-chain family member 4	0.48	< 0.001
Kpna3	karyopherin (importin) alpha 3	0.48	0.051
D10Ertd709e	DNA segment, Chr 10, ERATO Doi 709, expressed	0.48	< 0.001
Rnf13	ring finger protein 13	0.48	0.051
Dio2	deiodinase, iodothyronine, type II	0.48	< 0.001
Man1a2	mannosidase, alpha, class 1A, member 2	0.48	0.051
Lin7c	lin-7 homolog C (C. elegans)	0.48	0.051
Nat12	N-acetyltransferase 12	0.48	0.051
Braf	Braf transforming gene	0.48	< 0.001
Sp1	trans-acting transcription factor 1	0.48	< 0.001
Prps2	phosphoribosyl pyrophosphate synthetase 2	0.48	0.051
Hbp1	high mobility group box transcription factor 1	0.48	0.051
Ptprb	protein tyrosine phosphatase, receptor type, B	0.49	< 0.001
Arhgef2	rho/rac guanine nucleotide exchange factor (GEF) 2	0.49	0.051
Ints8	integrator complex subunit 8	0.49	0.051
Ednra	endothelin receptor type A	0.49	< 0.001
Rbbp4	retinoblastoma binding protein 4	0.49	< 0.001
Xiap	X-linked inhibitor of apoptosis	0.49	0.051
Lmnb1	lamin B1	0.49	< 0.001
Trib2	tribbles homolog 2 (Drosophila)	0.49	0.051
Pafah1b2	platelet-activating factor acetylhydrolase, isoform 1b, subunit 2	0.49	< 0.001
Rps6kb1	ribosomal protein S6 kinase, polypeptide 1	0.49	0.051
Pcnx	pecanex homolog (Drosophila)	0.49	0.051
Otud4	OTU domain containing 4	0.49	0.051
Etnk1	ethanolamine kinase 1	0.49	0.051

Cxcl1	chemokine (C-X-C motif) ligand 1	0.49	0.051
Gnptab	N-acetylglucosamine-1-phosphate transferase, alpha and beta subunits	0.49	0.051
Ly6g6c	lymphocyte antigen 6 complex, locus G6C	0.49	0.051
Mier1	mesoderm induction early response 1 homolog (Xenopus laevis	0.49	0.051
Eif4h	eukaryotic translation initiation factor 4H	0.49	0.051
Sema3c	sema domain, immunoglobulin domain (Ig), short basic domain, secreted, (semaphorin) 3C	0.49	0.051
Cap1	CAP, adenylate cyclase-associated protein 1 (yeast)	0.49	0.051
1810030O07Rik	RIKEN cDNA 1810030O07 gene	0.49	0.051
5730416O20Rik	RIKEN cDNA 5730416O20 gene	0.49	0.051
Usp12	ubiquitin specific peptidase 12	0.50	< 0.001
Atp8a1	ATPase, aminophospholipid transporter (APLT), class I, type 8A, member 1	0.50	< 0.001
Chn1	chimerin (chimaerin) 1	0.50	< 0.001
Rrm2b	ribonucleotide reductase M2 B (TP53 inducible)	0.50	< 0.001
Appl1	adaptor protein, phosphotyrosine interaction, PH domain and leucine zipper containing 1	0.50	< 0.001
Adam10	a disintegrin and metallopeptidase domain 10	0.50	0.051
Dapp1	dual adaptor for phosphotyrosine and 3-phosphoinositides 1	0.50	0.051
Cpd	carboxypeptidase D	0.50	0.051
6720457D02Rik	RIKEN cDNA 6720457D02 gene	2.01	< 0.001
Pgm2l1	phosphoglucomutase 2-like 1	2.01	< 0.001
Slco4c1	solute carrier organic anion transporter family, member 4C1	2.01	< 0.001
Zc3h15	zinc finger CCCH-type containing 15	2.02	< 0.001
Trmt6	tRNA methyltransferase 6 homolog (S. cerevisiae)	2.02	< 0.001
Nhsl2	NHS-like 2	2.02	< 0.001
Ankrd11	ankyrin repeat domain 11	2.02	< 0.001
Zfp292	zinc finger protein 292	2.03	< 0.001
Mical2	microtubule associated monoxygenase, calponin and LIM domain containing 2	2.03	< 0.001
Pisd-ps3	phosphatidylserine decarboxylase, pseudogene 3	2.04	< 0.001
Phka2	phosphorylase kinase alpha 2	2.04	< 0.001
Nup214	nucleoporin 214	2.04	< 0.001
6820402A03Rik	RIKEN cDNA 6820402A03 gene	2.04	< 0.001
Hnrnpu	heterogeneous nuclear ribonucleoprotein U	2.05	< 0.001
Gatad2b	GATA zinc finger domain containing 2B	2.06	< 0.001
A330076H08Rik	RIKEN cDNA A330076H08 gene	2.06	< 0.001
9430047G12Rik	RIKEN cDNA 9430047G12 gene	2.06	< 0.001
Fubp1	far upstream element (FUSE) binding protein 1	2.07	< 0.001
Wdfy3	WD repeat and FYVE domain containing 3	2.09	< 0.001
Cdkn1b	cyclin-dependent kinase inhibitor 1B	2.09	< 0.001
4933411K20Rik	RIKEN cDNA 4933411K20 gene	2.10	< 0.001
Kank2	KN motif and ankyrin repeat domains 2	2.10	< 0.001
Vps13a	vacuolar protein sorting 13A (yeast)	2.11	< 0.001
Lrrk1	leucine-rich repeat kinase 1	2.12	< 0.001
Iqgap1	IQ motif containing GTPase activating protein 1	2.12	< 0.001
Gan	giant axonal neuropathy	2.13	< 0.001
Nr2f2	nuclear receptor subfamily 2, group F, member 2	2.13	< 0.001
Usp37	ubiquitin specific peptidase 37	2.13	< 0.001

Eif2c3	eukaryotic translation initiation factor 2C, 3	2.15	< 0.001
Zbtb7a	zinc finger and BTB domain containing 7a	2.15	< 0.001
Zfp207	zinc finger protein 207	2.16	< 0.001
Rapgef6	Rap guanine nucleotide exchange factor (GEF) 6	2.17	< 0.001
Snapc5	small nuclear RNA activating complex, polypeptide 5	2.17	< 0.001
4933407K13Rik	RIKEN cDNA 4933407K13 gene	2.18	< 0.001
Sord	sorbitol dehydrogenase	2.18	< 0.001
Tle4	transducin-like enhancer of split 4, homolog of Drosophila E(spl)	2.18	0.085
Rbm26	RNA binding motif protein 26	2.18	< 0.001
6430548M08Rik	RIKEN cDNA 6430548M08 gene	2.19	< 0.001
4833420G17Rik	RIKEN cDNA 4833420G17 gene	2.20	< 0.001
Casc4	cancer susceptibility candidate 4	2.21	< 0.001
Ammecr1	Alport syndrome, mental retardation, midface hypoplasia and elliptocytosis chromosomal region gene 1 homolog (human)	2.21	< 0.001
A930038C07Rik	RIKEN cDNA A930038C07 gene	2.22	< 0.001
Gria3	glutamate receptor, ionotropic, AMPA3 (alpha 3)	2.22	< 0.001
Fam117b	family with sequence similarity 117, member B	2.22	< 0.001
Sfi1	Sfi1 homolog, spindle assembly associated (yeast)	2.24	< 0.001
Rap2b	RAP2B, member of RAS oncogene family	2.24	< 0.001
Epn2	epsin 2	2.24	< 0.001
AU018466	expressed sequence AU018466	2.25	< 0.001
Sema6d	sema domain, transmembrane domain (TM), and cytoplasmic domain, (semaphorin) 6D	2.25	0.085
Zbtb16	zinc finger and BTB domain containing 16	2.26	< 0.001
Atp11c	ATPase, class VI, type 11C	2.26	< 0.001
C78505	expressed sequence C78505	2.27	< 0.001
Rian	RNA imprinted and accumulated in nucleus	2.27	0.085
Arrdc3	arrestin domain containing 3	2.28	< 0.001
C77405	expressed sequence C77405	2.29	< 0.001
Ccrl1	chemokine (C-C motif) receptor-like 1	2.32	0.085
Cml3	camello-like 3	2.33	< 0.001
Gm10883	predicted gene 10883	2.33	< 0.001
Gnal	guanine nucleotide binding protein, alpha stimulating, olfactory type	2.33	< 0.001
Clec16a	C-type lectin domain family 16, member A	2.33	< 0.001
E130012A19Rik	RIKEN cDNA E130012A19 gene	2.33	< 0.001
Scaper	S phase cyclin A-associated protein in the ER	2.37	< 0.001
D5Ertd579e	DNA segment, Chr 5, ERATO Doi 579, expressed	2.38	< 0.001
Malat1	metastasis associated lung adenocarcinoma transcript 1 (non-coding RNA)	2.38	< 0.001
C80425	expressed sequence C80425	2.39	< 0.001
Nnt	nicotinamide nucleotide transhydrogenase	2.40	< 0.001
Crkrs	CDC2-related kinase, arginine/serine-rich	2.41	< 0.001
Usp15	ubiquitin specific peptidase 15	2.41	< 0.001
Acbd3	acyl-Coenzyme A binding domain containing 3	2.42	< 0.001
Zfx	zinc finger protein X-linked	2.42	< 0.001
Mbnl1	muscleblind-like 1 (Drosophila)	2.45	< 0.001
Dnmt1	DNA methyltransferase (cytosine-5) 1	2.46	< 0.001
2610020P09Rik	RIKEN cDNA 2610020P09 gene	2.48	< 0.001

Ylpm1	YLP motif containing 1	2.49	< 0.001
Gabpb2	GA repeat binding protein, beta 2	2.49	< 0.001
Cys1	cystin 1	2.52	< 0.001
Dbp	D site albumin promoter binding protein	2.55	< 0.001
Baz2a	bromodomain adjacent to zinc finger domain, 2A	2.57	< 0.001
Plcb4	phospholipase C, beta 4	2.57	< 0.001
Tnrc18	trinucleotide repeat containing 18	2.58	< 0.001
Elk3	ELK3, member of ETS oncogene family	2.58	< 0.001
Fbxo32	F-box protein 32	2.59	< 0.001
Ngp	neutrophilic granule protein	2.59	0.085
Sfpq	splicing factor proline/glutamine rich (polypyrimidine tract binding protein associated)	2.63	< 0.001
Tdo2	tryptophan 2,3-dioxygenase	2.63	< 0.001
5830407P18Rik	RIKEN cDNA 5830407P18 gene	2.66	< 0.001
Trpm7	transient receptor potential cation channel, subfamily M, member 7	2.67	< 0.001
Cbx5	chromobox homolog 5 (Drosophila HP1a)	2.67	< 0.001
Vps4b	vacuolar protein sorting 4b (yeast)	2.68	< 0.001
BC005685	cDNA sequence BC005685	2.71	< 0.001
Igh	immunoglobulin heavy chain complex	2.73	< 0.001
Per3	period homolog 3 (Drosophila)	2.75	< 0.001
LOC667118	zinc finger, BED domain containing 4 pseudogene	2.77	< 0.001
Tef	thyrotroph embryonic factor	2.78	< 0.001
Hjurp	Holliday junction recognition protein	2.82	< 0.001
2610024D14Rik	RIKEN cDNA 2610024D14 gene	2.89	< 0.001
Stfa2l1	stefin A2 like 1	2.94	< 0.001
Zfp101	zinc finger protein 101	2.94	< 0.001
Utrn	utrophin	2.96	< 0.001
2610301F02Rik	RIKEN cDNA 2610301F02 gene	3.03	< 0.001
NA	NA	3.08	0.085
Cep110	centrosomal protein 110	3.16	< 0.001
Mga	MAX gene associated	3.30	< 0.001
Airn	antisense Igf2r RNA	3.33	< 0.001
4921513D23Rik	RIKEN cDNA 4921513D23 gene	3.34	< 0.001
Hlf	hepatic leukemia factor	3.34	< 0.001
Crebbp	CREB binding protein	3.40	< 0.001
Slc15a2	solute carrier family 15 (H+/peptide transporter), member 2	3.59	< 0.001
Cd163l1	CD163 molecule-like 1	3.61	< 0.001
Nckap1l	NCK associated protein 1 like	3.74	< 0.001
Mid1	midline 1	3.77	< 0.001
Dab2ip	disabled homolog 2 (Drosophila) interacting protein	3.81	< 0.001
Bat2d	BAT2 domain containing 1	3.94	< 0.001
Dnmt3a	DNA methyltransferase 3A	4.15	< 0.001
Plec1	plectin 1	5.21	< 0.001
Ighg	Immunoglobulin heavy chain (gamma polypeptide)	5.29	< 0.001
Igj	immunoglobulin joining chain	8.34	< 0.001

[a] FC: fold change, which is calculated by dividing the expression in VILI-exposed nmMLCK KO mice by the expression in WT VILI-exposed mice.
