# Supplementary material for: Genes Influenced by the Non-Muscle Isoform of Myosin Light Chain Kinase Impact Human Cancer Prognosis
Source: PLoS One. 2014 Apr 8;9(4):e94325. doi: 10.1371/journal.pone.0094325 (PMC3979809; doi:10.1371/journal.pone.0094325)
Supplement: Table S2 — Differentially expressed genes between WT control and VILI-exposed WT mice. (PDF) [file pone.0094325.s007.pdf]

Table S2. Differentially expressed genes between WT control and VILI-exposed WT mice

Gene symbol	Gene title	FC[a]	FDR (%)
Zfp467	zinc finger protein 467	0.05	0.0
Hspa1a	heat shock protein 1A	0.06	0.0
Hspa1b	heat shock protein 1B	0.06	0.0
Gm1337	predicted gene 1337	0.07	0.0
Gm15453 /// Rbm3	predicted gene 15453 /// RNA binding motif protein 3	0.09	0.0
Dbp	D site albumin promoter binding protein	0.10	0.0
Car3	carbonic anhydrase 3	0.13	0.0
Gm8267	predicted gene 8267	0.15	0.0
Foxj1	forkhead box J1	0.18	0.0
Sox18	SRY-box containing gene 18	0.19	0.0
Per3	Period homolog 3 (Drosophila)	0.19	0.0
2210020M01Rik	RIKEN cDNA 2210020M01 gene	0.19	0.0
Fbxo30	F-box protein 30	0.20	0.0
Klhdc5	kelch domain containing 5	0.20	0.0
Akap14	A kinase (PRKA) anchor protein 14	0.20	0.0
Fam13a	family with sequence similarity 13, member A	0.21	0.0
Klf2	Kruppel-like factor 2 (lung)	0.21	0.0
2810055F11Rik	RIKEN cDNA 2810055F11 gene	0.21	0.0
Cyp2a4 /// Cyp2a5	cytochrome P450, family 2, subfamily a, polypeptide 4 /// cytochrome P450, family 2, subfamily a, polypeptide 5	0.21	0.0
Thap2	THAP domain containing, apoptosis associated protein 2	0.22	0.0
Ggta1	glycoprotein galactosyltransferase alpha 1, 3	0.22	0.0
Rassf9	Ras association (RalGDS/AF-6) domain family (N-terminal) member 9	0.23	0.0
Ptger2	prostaglandin E receptor 2 (subtype EP2)	0.23	0.0
Zfp667	zinc finger protein 667	0.23	0.0
6720407P12Rik	RIKEN cDNA 6720407P12 gene	0.23	0.0
Ccdc25	coiled-coil domain containing 25	0.23	0.0
Tppp	tubulin polymerization promoting protein	0.23	0.0
Rreb1	ras responsive element binding protein 1	0.23	0.0
Ttc3	tetratricopeptide repeat domain 3	0.24	0.0
Myb	myeloblastosis oncogene	0.24	0.0
AU021034	expressed sequence AU021034	0.24	0.0
Gpr182	G protein-coupled receptor 182	0.24	0.0
Fam47e	family with sequence similarity 47, member E	0.24	0.0
A430108G06Rik	RIKEN cDNA A430108G06 gene	0.24	0.0
Capn5	calpain 5	0.25	0.0
2900006K08Rik	RIKEN cDNA 2900006K08 gene	0.25	0.0
D4Wsu53e	DNA segment, Chr 4, Wayne State University 53, expressed	0.26	0.0
4930451C15Rik	RIKEN cDNA 4930451C15 gene	0.26	0.0
Nck1	non-catalytic region of tyrosine kinase adaptor protein 1	0.26	0.0
Npy1r	neuropeptide Y receptor Y1	0.26	0.0
Calcrl	calcitonin receptor-like	0.26	0.0
Thsd1	thrombospondin, type I, domain 1	0.26	0.0
Tekt4	tektin 4	0.27	0.0

2210019G11Rik	RIKEN cDNA 2210019G11 gene	0.27	0.0
Kank4	KN motif and ankyrin repeat domains 4	0.27	0.0
Dcdc2b	doublecortin domain containing 2b	0.27	0.0
A330076H08Rik	RIKEN cDNA A330076H08 gene	0.27	0.0
Cml3	camello-like 3	0.27	0.0
Lrrc36	leucine rich repeat containing 36	0.27	0.0
Fads6	fatty acid desaturase domain family, member 6	0.27	0.0
Polk	polymerase (DNA directed), kappa	0.28	0.0
Ubxn10	UBX domain protein 10	0.28	0.0
H2-T24	histocompatibility 2, T region locus 24	0.28	0.0
A530020G20Rik	RIKEN cDNA A530020G20 gene	0.28	0.0
Atat1	alpha tubulin acetyltransferase 1	0.28	0.0
D630039A03Rik	RIKEN cDNA D630039A03 gene	0.29	0.0
Dnajb1	DnaJ (Hsp40) homolog, subfamily B, member 1	0.29	0.0
2010007H06Rik	RIKEN cDNA 2010007H06 gene	0.29	0.0
N4bp3	NEDD4 binding protein 3	0.29	0.0
Grtp1	GH regulated TBC protein 1	0.29	0.0
Meox2	mesenchyme homeobox 2	0.29	0.0
Stard9	START domain containing 9	0.29	0.0
4833427G06Rik	RIKEN cDNA 4833427G06 gene	0.29	0.0
Dyx1c1	dyslexia susceptibility 1 candidate 1 homolog (human)	0.29	0.0
Lrrc17	leucine rich repeat containing 17	0.29	0.0
Cys1	cystin 1	0.29	0.0
Hlf	hepatic leukemia factor	0.30	0.0
Kif26b	kinesin family member 26B	0.30	0.0
Ube2b	ubiquitin-conjugating enzyme E2B	0.30	0.0
Hspa12b	heat shock protein 12B	0.30	0.0
Zfp420	zinc finger protein 420	0.31	7.8
Agphd1	aminoglycoside phosphotransferase domain containing 1	0.31	0.0
Chchd7	Coiled-coil-helix-coiled-coil-helix domain containing 7	0.31	0.0
Uty	ubiquitously transcribed tetratricopeptide repeat gene, Y chromosome	0.31	0.0
Zbtb46	zinc finger and BTB domain containing 46	0.31	0.0
Senp7	SUMO1/sentrin specific peptidase 7	0.31	0.0
Cep70	centrosomal protein 70	0.31	0.0
Mxi1	Max interacting protein 1	0.31	0.0
Zfp760	zinc finger protein 760	0.31	0.0
Fgfr3	fibroblast growth factor receptor 3	0.31	0.0
Herc6	hect domain and RLD 6	0.31	0.0
Fhl1	four and a half LIM domains 1	0.32	0.0
Caps2	calcyphosphine 2	0.32	0.0
Hdac11	histone deacetylase 11	0.32	0.0
Prr18	proline rich region 18	0.32	0.0
1190005F20Rik	RIKEN cDNA 1190005F20 gene	0.32	0.0
Ezh1	enhancer of zeste homolog 1 (Drosophila)	0.32	0.0
Tcf21	transcription factor 21	0.32	0.0
Mtus2	microtubule associated tumor suppressor candidate 2	0.32	0.0
5430414B12Rik	RIKEN cDNA 5430414B12 gene	0.32	0.0
BB182297	expressed sequence BB182297	0.32	0.0
Fam55c	family with sequence similarity 55, member C	0.32	0.0

Igh-VJ558 /// Igha	immunoglobulin heavy chain (J558 family) /// immunoglobulin heavy constant alpha	0.33	7.8
6430706D22Rik /// A730008H23Rik /// Hjurp	RIKEN cDNA 6430706D22 gene /// RIKEN cDNA A730008H23 gene /// Holliday junction recognition protein	0.33	0.0
Wdr19	WD repeat domain 19	0.33	0.0
Zxdc	ZXD family zinc finger C	0.33	2.8
9130015G15Rik	RIKEN cDNA 9130015G15 gene	0.33	0.0
2810055G20Rik	RIKEN cDNA 2810055G20 gene	0.33	0.0
Fancc	Fanconi anemia, complementation group C	0.33	0.0
Trim14	tripartite motif-containing 14	0.34	0.0
Prrx1	paired related homeobox 1	0.34	0.0
Agbl5	ATP/GTP binding protein-like 5	0.34	0.0
Kcna2	potassium voltage-gated channel, shaker-related subfamily, member 2	0.34	0.0
Pla2g15	phospholipase A2, group XV	0.34	0.0
E130308A19Rik	RIKEN cDNA E130308A19 gene	0.34	0.0
Dusp19	dual specificity phosphatase 19	0.34	2.8
Mdh1b	malate dehydrogenase 1B, NAD (soluble)	0.34	0.0
Ints3	integrator complex subunit 3	0.34	0.0
Cd59a	CD59a antigen	0.34	0.0
Mettl8	methyltransferase like 8	0.34	0.0
4932425I24Rik	RIKEN cDNA 4932425I24 gene	0.35	0.0
Map3k12	mitogen-activated protein kinase kinase kinase 12	0.35	0.0
Rps6ka5	ribosomal protein S6 kinase, polypeptide 5	0.35	0.0
4833442J19Rik	RIKEN cDNA 4833442J19 gene	0.35	0.0
Rab36	RAB36, member RAS oncogene family	0.35	0.0
Plekha2	pleckstrin homology domain-containing, family A (phosphoinositide binding specific) member 2	0.35	0.0
Tead2	TEA domain family member 2	0.35	0.0
Dixdc1	DIX domain containing 1	0.35	0.0
Fmo5	flavin containing monooxygenase 5	0.36	0.0
Zfp54	zinc finger protein 54	0.36	0.0
Bivm	basic, immunoglobulin-like variable motif containing	0.36	0.0
6330439K17Rik	RIKEN cDNA 6330439K17 gene	0.36	0.0
Snapc3	small nuclear RNA activating complex, polypeptide 3	0.36	0.0
Ripply3	ripply3 homolog (zebrafish)	0.36	0.0
Asb14	ankyrin repeat and SOCS box-containing 14	0.36	0.0
Tet1	tet methylcytosine dioxygenase 1	0.36	0.0
Stk33	serine/threonine kinase 33	0.37	0.0
Nek1	NIMA (never in mitosis gene a)-related expressed kinase 1	0.37	0.0
Smad6	SMAD family member 6	0.37	0.0
Rsph4a	radial spoke head 4 homolog A (Chlamydomonas)	0.37	0.0
Tfb1m	transcription factor B1, mitochondrial	0.37	0.0
Ces1f	carboxylesterase 1F	0.37	0.0
Wdyhv1	WDYHV motif containing 1	0.37	0.0
Rab38	RAB38, member of RAS oncogene family	0.37	0.0
Rasgef1a	RasGEF domain family, member 1A	0.37	0.0
Dtna	dystrobrevin alpha	0.37	0.0

Ak3	adenylate kinase 3	0.37	0.0
Gm129	predicted gene 129	0.37	0.0
Nr1d2	nuclear receptor subfamily 1, group D, member 2	0.38	0.0
Tnfsf10	tumor necrosis factor (ligand) superfamily, member 10	0.38	2.8
C80889	expressed sequence C80889	0.38	0.0
Hist3h2a	histone cluster 3, H2a	0.38	0.0
Amigo2	adhesion molecule with Ig like domain 2	0.38	0.0
Ttc21b	tetratricopeptide repeat domain 21B	0.38	0.0
Zfp934	zinc finger protein 934	0.38	0.0
Slc15a2	solute carrier family 15 (H+/peptide transporter), member 2	0.38	2.8
Nudt7	nudix (nucleoside diphosphate linked moiety X)-type motif 7	0.38	0.0
9430047G12Rik	RIKEN cDNA 9430047G12 gene	0.38	0.0
Wrn	Werner syndrome homolog (human)	0.38	0.0
Kctd21	potassium channel tetramerisation domain containing 21	0.38	0.0
Calhm2	calcium homeostasis modulator 2	0.38	0.0
Gm5595	predicted gene 5595	0.38	0.0
A130004G11Rik	RIKEN cDNA A130004G11 gene	0.38	0.0
Tslp	thymic stromal lymphopoietin	0.38	0.0
Plscr4	phospholipid scramblase 4	0.38	0.0
A730054J21Rik	RIKEN cDNA A730054J21 gene	0.38	0.0
Kank3	KN motif and ankyrin repeat domains 3	0.39	0.0
Rarg	retinoic acid receptor, gamma	0.39	0.0
Pknox2	Pbx/knotted 1 homeobox 2	0.39	0.0
Il17rd	interleukin 17 receptor D	0.39	0.0
Ankrd26	ankyrin repeat domain 26	0.39	0.0
Lyl1	lymphoblastomic leukemia 1	0.39	0.0
Efcab1	EF hand calcium binding domain 1	0.39	0.0
Edc3	enhancer of mRNA decapping 3 homolog (S. cerevisiae)	0.39	0.0
Phf21a	PHD finger protein 21A	0.39	0.0
Lrrc51	leucine rich repeat containing 51	0.39	0.0
Tmem206	transmembrane protein 206	0.39	2.8
Hist3h2a /// Trim17	histone cluster 3, H2a /// tripartite motif-containing 17	0.39	0.0
Yes1	Yamaguchi sarcoma viral (v-yes) oncogene homolog 1	0.39	0.0
Il15	interleukin 15	0.39	0.0
G0s2	G0/G1 switch gene 2	0.39	0.0
Mns1	meiosis-specific nuclear structural protein 1	0.39	0.0
AA386476 /// LOC100504975	expressed sequence AA386476 /// uncharacterized LOC100504975	0.40	0.0
Arhgap12	Rho GTPase activating protein 12	0.40	0.0
Dnmt3a	DNA methyltransferase 3A	0.40	0.0
Hs1bp3	HCLS1 binding protein 3	0.40	0.0
9430020K01Rik	RIKEN cDNA 9430020K01 gene	0.40	0.0
Slc46a3	solute carrier family 46, member 3	0.40	0.0
Ip6k2	inositol hexaphosphate kinase 2	0.40	0.0
Trp53bp1	transformation related protein 53 binding protein 1	0.40	2.8
Zfp846	zinc finger protein 846	0.40	0.0
Cckar	cholecystokinin A receptor	0.40	0.0
Cdon	cell adhesion molecule-related/down-regulated by	0.40	0.0

	oncogenes		
Cd97	CD97 antigen	0.40	0.0
1700013F07Rik	RIKEN cDNA 1700013F07 gene	0.40	0.0
Nab2	Ngfi-A binding protein 2	0.40	2.8
Card10	caspase recruitment domain family, member 10	0.40	0.0
Klhl24	kelch-like 24 (Drosophila)	0.40	0.0
Lpar3	lysophosphatidic acid receptor 3	0.40	0.0
Six5	sine oculis-related homeobox 5 homolog (Drosophila)	0.40	0.0
Zfp82	zinc finger protein 82	0.40	7.8
C77648	expressed sequence C77648	0.40	0.0
Sigmar1	sigma non-opioid intracellular receptor 1	0.41	2.8
Wdr78	WD repeat domain 78	0.41	0.0
Dynlt1f	dynein light chain Tctex-type 1F	0.41	0.0
Ccdc141	coiled-coil domain containing 141	0.41	7.8
Gemin8	gem (nuclear organelle) associated protein 8	0.41	0.0
Senp8	SUMO/sentrin specific peptidase 8	0.41	2.8
Ttc14	tetratricopeptide repeat domain 14	0.41	0.0
Purg	purine-rich element binding protein G	0.41	0.0
Frem1	Fras1 related extracellular matrix protein 1	0.41	0.0
D730048J04Rik	RIKEN cDNA D730048J04 gene	0.41	0.0
Ganc	glucosidase, alpha; neutral C	0.41	0.0
Shisa2	shisa homolog 2 (Xenopus laevis)	0.41	0.0
Zfp790	zinc finger protein 790	0.41	0.0
Gm5918	predicted gene 5918	0.41	0.0
Glis2	GLIS family zinc finger 2	0.41	0.0
Ccdc28b	coiled coil domain containing 28B	0.41	0.0
Cc2d2a	coiled-coil and C2 domain containing 2A	0.41	0.0
BC046404	cDNA sequence BC046404	0.41	7.8
Eef2k	eukaryotic elongation factor-2 kinase	0.41	0.0
Wscd1	WSC domain containing 1	0.41	0.0
Grrp1	glycine/arginine rich protein 1	0.42	0.0
1700049G17Rik	RIKEN cDNA 1700049G17 gene	0.42	0.0
Ccdc30	coiled-coil domain containing 30	0.42	0.0
Rnf186	ring finger protein 186	0.42	0.0
Sox17	SRY-box containing gene 17	0.42	0.0
LOC100861749 /// Sfi1	protein SFI1 homolog /// Sfi1 homolog, spindle assembly associated (yeast)	0.42	0.0
Nudcd1	NudC domain containing 1	0.42	0.0
Trim37	tripartite motif-containing 37	0.42	0.0
Rab2b	RAB2B, member RAS oncogene family	0.42	0.0
Zfp266	zinc finger protein 266	0.42	0.0
Aspa	aspartoacylase	0.42	0.0
Fgfr4	fibroblast growth factor receptor 4	0.42	0.0
Hibch	3-hydroxyisobutyryl-Coenzyme A hydrolase	0.42	0.0
Per2	period homolog 2 (Drosophila)	0.42	0.0
Dnali1	dynein, axonemal, light intermediate polypeptide 1	0.42	0.0
Zfp524	zinc finger protein 524	0.42	0.0
Gm11944	predicted gene 11944	0.42	0.0
Pcdh18	protocadherin 18	0.42	0.0
Sord	sorbitol dehydrogenase	0.42	0.0

Fzd7	frizzled homolog 7 (Drosophila)	0.42	0.0
Gm626	predicted gene 626	0.42	0.0
Cep350	centrosomal protein 350	0.42	0.0
Vps13a	vacuolar protein sorting 13A (yeast)	0.43	0.0
4922501C03Rik	RIKEN cDNA 4922501C03 gene	0.43	0.0
Dleu2	deleted in lymphocytic leukemia, 2	0.43	2.8
Hyal1 /// Nat6	hyaluronoglucosaminidase 1 /// N-acetyltransferase 6	0.43	0.0
Igj	immunoglobulin joining chain	0.43	0.0
Gulp1	GULP, engulfment adaptor PTB domain containing 1	0.43	0.0
Adamts20	a disintegrin-like and metallopeptidase (reprolysin type) with thrombospondin type 1 motif, 20	0.43	0.0
Rnf135	ring finger protein 135	0.43	0.0
Xiap	X-linked inhibitor of apoptosis	0.43	2.8
Hsph1	heat shock 105kDa/110kDa protein 1	0.43	0.0
Baz1b	bromodomain adjacent to zinc finger domain, 1B	0.43	0.0
Jam2	junction adhesion molecule 2	0.43	0.0
Invs	inversin	0.43	0.0
Plch1	phospholipase C, eta 1	0.43	0.0
Nuak1	NUAK family, SNF1-like kinase, 1	0.43	0.0
Cthrc1	collagen triple helix repeat containing 1	0.43	2.8
Slc25a27	Solute carrier family 25, member 27	0.43	0.0
1700029J07Rik	RIKEN cDNA 1700029J07 gene	0.43	0.0
Gm19935	predicted gene, 19935	0.43	0.0
Ephb4	Eph receptor B4	0.43	0.0
Gm19439	predicted gene, 19439	0.43	0.0
1810063B05Rik	RIKEN cDNA 1810063B05 gene	0.43	0.0
Csnk2a1	casein kinase 2, alpha 1 polypeptide	0.43	0.0
Nkd2	naked cuticle 2 homolog (Drosophila)	0.43	0.0
2900092E17Rik	RIKEN cDNA 2900092E17 gene	0.43	0.0
Gga2	golgi associated, gamma adaptin ear containing, ARF binding protein 2	0.44	0.0
Efhc1	EF-hand domain (C-terminal) containing 1	0.44	0.0
Fbxl20	F-box and leucine-rich repeat protein 20	0.44	0.0
Spice1	spindle and centriole associated protein 1	0.44	0.0
Scaper	S phase cyclin A-associated protein in the ER	0.44	0.0
Atf7ip	activating transcription factor 7 interacting protein	0.44	0.0
Nudt21	nudix (nucleoside diphosphate linked moiety X)-type motif 21	0.44	7.8
1110017F19Rik	RIKEN cDNA 1110017F19 gene	0.44	0.0
Tbc1d24	TBC1 domain family, member 24	0.44	0.0
Klc1	kinesin light chain 1	0.44	0.0
Atf2 /// LOC100047997	activating transcription factor 2 /// cyclic AMP-dependent transcription factor ATF-2-like	0.44	0.0
Khnyn	KH and NYN domain containing	0.44	0.0
Cep68	centrosomal protein 68	0.44	0.0
Angpt1	angiopoietin 1	0.44	0.0
1810012P15Rik	RIKEN cDNA 1810012P15 gene	0.44	0.0
Nudt5	nudix (nucleoside diphosphate linked moiety X)-type motif 5	0.44	0.0
Chia	chitinase, acidic	0.44	0.0

Fam120b	family with sequence similarity 120, member B	0.44	0.0
Robo2	roundabout homolog 2 (Drosophila)	0.44	2.8
Chd9	chromodomain helicase DNA binding protein 9	0.44	0.0
Zfp248	zinc finger protein 248	0.44	2.8
Etv1	ets variant gene 1	0.44	0.0
Traf1	TNF receptor-associated factor 1	0.44	0.0
Ttc29	tetratricopeptide repeat domain 29	0.44	0.0
C2cd2	C2 calcium-dependent domain containing 2	0.44	0.0
2810410L24Rik	RIKEN cDNA 2810410L24 gene	0.44	0.0
Flt3l /// Rpl13a	FMS-like tyrosine kinase 3 ligand /// ribosomal protein L13A	0.44	0.0
Ikzf2	IKAROS family zinc finger 2	0.45	0.0
1700094D03Rik	RIKEN cDNA 1700094D03 gene	0.45	0.0
Sh3d19	SH3 domain protein D19	0.45	0.0
Synpo2	synaptopodin 2	0.45	7.8
Hs6st2	heparan sulfate 6-O-sulfotransferase 2	0.45	0.0
4930420K17Rik	RIKEN cDNA 4930420K17 gene	0.45	0.0
Tfcp2	transcription factor CP2	0.45	0.0
Ttc9c	tetratricopeptide repeat domain 9C	0.45	2.8
Gja4	gap junction protein, alpha 4	0.45	0.0
Rhebl1	Ras homolog enriched in brain like 1	0.45	0.0
Plcb3	phospholipase C, beta 3	0.45	0.0
Ptgfr	prostaglandin F receptor	0.45	0.0
2900046F13Rik	RIKEN cDNA 2900046F13 gene	0.45	0.0
Mynn	myoneurin	0.45	0.0
Golph3l	golgi phosphoprotein 3-like	0.45	0.0
C920008N22Rik	RIKEN cDNA C920008N22 gene	0.45	0.0
Yeats2	YEATS domain containing 2	0.45	0.0
Aass	aminoadipate-semialdehyde synthase	0.45	0.0
Trim34a /// Trim34b	tripartite motif-containing 34A /// tripartite motif-containing 34B	0.45	0.0
Cenpj	centromere protein J	0.45	0.0
Dzip1l	DAZ interacting protein 1-like	0.45	0.0
Thap3	THAP domain containing, apoptosis associated protein 3	0.45	0.0
Mdm4	transformed mouse 3T3 cell double minute 4	0.45	0.0
4933411K20Rik	RIKEN cDNA 4933411K20 gene	0.45	0.0
Fert2	fer (fms/fps related) protein kinase, testis specific 2	0.45	0.0
Elmod1	ELMO domain containing 1	0.45	0.0
Fam82b	family with sequence similarity 82, member B	0.45	0.0
Crebzf	CREB/ATF bZIP transcription factor	0.45	0.0
Lrrc6	leucine rich repeat containing 6 (testis)	0.45	0.0
2410002O22Rik	RIKEN cDNA 2410002O22 gene	0.45	0.0
Sirt5	sirtuin 5 (silent mating type information regulation 2 homolog) 5 (S. cerevisiae)	0.45	0.0
Ubxn7	UBX domain protein 7	0.46	2.8
Akr1c19	aldo-keto reductase family 1, member C19	0.46	0.0
5031426D15Rik	RIKEN cDNA 5031426D15 gene	0.46	0.0
Ints6	integrator complex subunit 6	0.46	2.8
E430024I08Rik	RIKEN cDNA E430024I08 gene	0.46	0.0
D4Bwg0951e	DNA segment, Chr 4, Brigham & Women's Genetics 0951	0.46	0.0

	expressed		
Pisd-ps1 /// Pisd-ps3	phosphatidylserine decarboxylase, pseudogene 1 /// phosphatidylserine decarboxylase, pseudogene 3	0.46	0.0
Zfp14	zinc finger protein 14	0.46	0.0
Dnahc12	dynein, axonemal, heavy chain 12	0.46	0.0
Pgpep1	pyroglutamyl-peptidase I	0.46	0.0
Ints8	integrator complex subunit 8	0.46	0.0
Scai	suppressor of cancer cell invasion	0.46	0.0
Papss2	3'-phosphoadenosine 5'-phosphosulfate synthase 2	0.46	2.8
Slc38a6	solute carrier family 38, member 6	0.46	0.0
Itsn1	intersectin 1 (SH3 domain protein 1A)	0.46	0.0
Zmat1	zinc finger, matrin type 1	0.46	0.0
Gm10374	predicted gene 10374	0.46	0.0
Zfp260	zinc finger protein 260	0.46	0.0
Rbm5	RNA binding motif protein 5	0.46	0.0
Vps33a	vacuolar protein sorting 33A (yeast)	0.46	0.0
Esyt3	extended synaptotagmin-like protein 3	0.46	0.0
1110032A04Rik	RIKEN cDNA 1110032A04 gene	0.46	0.0
Lbh	limb-bud and heart	0.46	0.0
9230114K14Rik	RIKEN cDNA 9230114K14 gene	0.46	0.0
Dcun1d2	DCN1, defective in cullin neddylation 1, domain containing 2 (S. cerevisiae)	0.46	2.8
Hey1	hairy/enhancer-of-split related with YRPW motif 1	0.46	0.0
Bcl11a	B cell CLL/lymphoma 11A (zinc finger protein)	0.46	7.8
1810011O10Rik	RIKEN cDNA 1810011O10 gene	0.46	0.0
Ptprz1	protein tyrosine phosphatase, receptor type Z, polypeptide 1	0.46	7.8
Tcap	titin-cap	0.46	2.8
Rasal2	RAS protein activator like 2	0.46	0.0
2610507I01Rik	RIKEN cDNA 2610507I01 gene	0.46	0.0
Lca5	Leber congenital amaurosis 5 (human)	0.46	0.0
Chst7	carbohydrate (N-acetylglucosamino) sulfotransferase 7	0.46	0.0
Gpt2	glutamic pyruvate transaminase (alanine aminotransferase) 2	0.46	2.8
Olfml1	olfactomedin-like 1	0.46	0.0
Slc6a2	solute carrier family 6 (neurotransmitter transporter, noradrenalin), member 2	0.46	0.0
Rab3ip	RAB3A interacting protein	0.46	0.0
Atic	5-aminoimidazole-4-carboxamide ribonucleotide formyltransferase/IMP cyclohydrolase	0.47	0.0
Stx3	syntaxin 3	0.47	0.0
Tardbp	TAR DNA binding protein	0.47	0.0
Chordc1	cysteine and histidine-rich domain (CHORD)-containing, zinc-binding protein 1	0.47	0.0
Cpeb1	cytoplasmic polyadenylation element binding protein 1	0.47	0.0
Dusp18	dual specificity phosphatase 18	0.47	0.0
Ckmt1	creatine kinase, mitochondrial 1, ubiquitous	0.47	2.8
Stat1	signal transducer and activator of transcription 1	0.47	0.0
Sept8	septin 8	0.47	0.0
Rph3al	rabphilin 3A-like (without C2 domains)	0.47	0.0

6820402A03Rik	RIKEN cDNA 6820402A03 gene	0.47	0.0
Mtcp1	mature T cell proliferation 1	0.47	0.0
Dzip1	DAZ interacting protein 1	0.47	0.0
Slc6a15	solute carrier family 6 (neurotransmitter transporter), member 15	0.47	0.0
Nqo2	NAD(P)H dehydrogenase, quinone 2	0.47	2.8
Caprin1	cell cycle associated protein 1	0.47	0.0
Dnaja4	DnaJ (Hsp40) homolog, subfamily A, member 4	0.47	0.0
Nr1h3	nuclear receptor subfamily 1, group H, member 3	0.47	2.8
Wdr96	WD repeat domain 96	0.47	0.0
Gpd1l	glycerol-3-phosphate dehydrogenase 1-like	0.47	0.0
Rarb	retinoic acid receptor, beta	0.47	0.0
She	src homology 2 domain-containing transforming protein E	0.47	0.0
Nnt	nicotinamide nucleotide transhydrogenase	0.47	0.0
Fgf7	fibroblast growth factor 7	0.47	0.0
Grasp	GRP1 (general receptor for phosphoinositides 1)-associated scaffold protein	0.47	0.0
Ogt	O-linked N-acetylglucosamine (GlcNAc) transferase (UDP-N-acetylglucosamine:polypeptide-N-acetylglucosaminyl transferase)	0.47	0.0
Gm20559	predicted gene, 20559	0.47	0.0
Hhip	Hedgehog-interacting protein	0.47	0.0
Fam46c	family with sequence similarity 46, member C	0.47	7.8
Slc4a1	solute carrier family 4 (anion exchanger), member 1	0.47	0.0
2900072G11Rik	RIKEN cDNA 2900072G11 gene	0.47	0.0
Csrnp2	cysteine-serine-rich nuclear protein 2	0.47	2.8
Gm12942 /// Zmym6	predicted gene 12942 /// zinc finger, MYM-type 6	0.47	0.0
Pank1	pantothenate kinase 1	0.47	0.0
Cdkn2c	cyclin-dependent kinase inhibitor 2C (p18, inhibits CDK4)	0.47	0.0
Osbpl6	oxysterol binding protein-like 6	0.47	0.0
Tdp1	tyrosyl-DNA phosphodiesterase 1	0.47	0.0
Hoxa5	homeobox A5	0.47	0.0
Dnaja1	DnaJ (Hsp40) homolog, subfamily A, member 1	0.47	0.0
Ccng2	cyclin G2	0.47	0.0
Inpp5e	inositol polyphosphate-5-phosphatase E	0.47	0.0
Ccdc162	coiled-coil domain containing 162	0.47	0.0
Six4	sine oculis-related homeobox 4 homolog (Drosophila)	0.47	0.0
Zfp60	zinc finger protein 60	0.47	0.0
Ddo	D-aspartate oxidase	0.47	0.0
Sytl4	synaptotagmin-like 4	0.47	0.0
Gm16136	predicted gene 16136	0.47	0.0
Mapk12	mitogen-activated protein kinase 12	0.47	0.0
Stmn2	stathmin-like 2	0.48	0.0
Tril	TLR4 interactor with leucine-rich repeats	0.48	0.0
Prkar2b	protein kinase, cAMP dependent regulatory, type II beta	0.48	0.0
6330403M23Rik /// Pura	RIKEN cDNA 6330403M23 gene /// purine rich element binding protein A	0.48	0.0
Zfp161	zinc finger protein 161	0.48	0.0
Rnf141	ring finger protein 141	0.48	0.0

Samd5	sterile alpha motif domain containing 5	0.48	2.8
Mlh3	mutL homolog 3 (E coli)	0.48	0.0
Mfsd9	major facilitator superfamily domain containing 9	0.48	0.0
Id1	inhibitor of DNA binding 1	0.48	0.0
2210018M11Rik	RIKEN cDNA 2210018M11 gene	0.48	0.0
Casp12	caspase 12	0.48	0.0
Gse1	genetic suppressor element 1	0.48	0.0
Tmeff1	transmembrane protein with EGF-like and two follistatin-like domains 1	0.48	0.0
Lrrn3	leucine rich repeat protein 3, neuronal	0.48	2.8
Skp2	S-phase kinase-associated protein 2 (p45)	0.48	7.8
Dzip3	DAZ interacting protein 3, zinc finger	0.48	0.0
Btbd3	BTB (POZ) domain containing 3	0.48	0.0
Tjp3	tight junction protein 3	0.48	0.0
Bpifa1	BPI fold containing family A, member 1	0.48	7.8
Rasgrp2	RAS, guanyl releasing protein 2	0.48	0.0
Pex26	peroxisomal biogenesis factor 26	0.48	0.0
4930430F08Rik	RIKEN cDNA 4930430F08 gene	0.48	0.0
Tatdn3	TatD DNase domain containing 3	0.48	0.0
Dnmt1	DNA methyltransferase (cytosine-5) 1	0.48	0.0
Mbtps1	membrane-bound transcription factor peptidase, site 1	0.48	7.8
Zscan22	zinc finger and SCAN domain containing 22	0.48	0.0
Map4k3	Mitogen-activated protein kinase kinase kinase kinase 3	0.48	0.0
Mtrr	5-methyltetrahydrofolate-homocysteine methyltransferase reductase	0.48	0.0
Ssx2ip	synovial sarcoma, X breakpoint 2 interacting protein	0.48	0.0
Cpt1c	carnitine palmitoyltransferase 1c	0.48	0.0
Ccdc45	coiled-coil domain containing 45	0.48	0.0
Efcab7	EF-hand calcium binding domain 7	0.48	0.0
Tecpr2	tectonin beta-propeller repeat containing 2	0.48	0.0
Myo9a	myosin IXa	0.48	0.0
Lsm10	U7 snRNP-specific Sm-like protein LSM10	0.48	0.0
Tmem116	transmembrane protein 116	0.48	7.8
Runx1t1	runt-related transcription factor 1; translocated to, 1 (cyclin D-related)	0.48	0.0
Aqp4	aquaporin 4	0.48	0.0
Rgs3	regulator of G-protein signaling 3	0.48	0.0
Tex11	testis expressed gene 11	0.48	0.0
Gfm2	G elongation factor, mitochondrial 2	0.48	0.0
Stxbp5	syntaxin binding protein 5 (tomosyn)	0.48	0.0
Traf3ip1	TRAF3 interacting protein 1	0.48	0.0
Prps2	phosphoribosyl pyrophosphate synthetase 2	0.48	2.8
Zfp708	zinc finger protein 708	0.49	0.0
2610034B18Rik	RIKEN cDNA 2610034B18 gene	0.49	0.0
Kit	kit oncogene	0.49	0.0
Ankmy2	ankyrin repeat and MYND domain containing 2	0.49	0.0
Ccdc85b	coiled-coil domain containing 85B	0.49	0.0
Fhdc1	FH2 domain containing 1	0.49	0.0
Cpsf6	cleavage and polyadenylation specific factor 6	0.49	0.0
9530067D14Rik	Riken cDNA 9530067D14 gene	0.49	0.0

Fam149a	family with sequence similarity 149, member A	0.49	0.0
1110021J02Rik	RIKEN cDNA 1110021J02 gene	0.49	0.0
Suv420h2	suppressor of variegation 4-20 homolog 2 (Drosophila)	0.49	0.0
Dguok	deoxyguanosine kinase	0.49	0.0
C87436	expressed sequence C87436	0.49	0.0
Cbx7	chromobox 7	0.49	0.0
Podxl	podocalyxin-like	0.49	2.8
Acox2	acyl-Coenzyme A oxidase 2, branched chain	0.49	0.0
Sp1	trans-acting transcription factor 1	0.49	0.0
Megf6	multiple EGF-like-domains 6	0.49	0.0
2900056M20Rik	RIKEN cDNA 2900056M20 gene	0.49	0.0
Rsph10b2	radial spoke head 10 homolog B (Chlamydomonas)	0.49	0.0
9530010C24Rik	RIKEN cDNA 9530010C24 gene	0.49	0.0
9330151L19Rik	RIKEN cDNA 9330151L19 gene	0.49	0.0
Tmcc2	transmembrane and coiled-coil domains 2	0.49	0.0
Tef	thyrotroph embryonic factor	0.49	0.0
Rad1	RAD1 homolog (S. pombe)	0.49	2.8
Dnaja3	DnaJ (Hsp40) homolog, subfamily A, member 3	0.49	0.0
Ttc30a1	tetratricopeptide repeat domain 30A1	0.49	0.0
Sspn	sarcospan	0.49	0.0
6330503K22Rik	RIKEN cDNA 6330503K22 gene	0.49	0.0
Tmem129	transmembrane protein 129	0.49	0.0
Chd6	chromodomain helicase DNA binding protein 6	0.49	0.0
Btrc	beta-transducin repeat containing protein	0.49	0.0
Asxl2	additional sex combs like 2 (Drosophila)	0.49	0.0
Irgm2	immunity-related GTPase family M member 2	0.49	0.0
Rgl3	ral guanine nucleotide dissociation stimulator-like 3	0.49	0.0
Mthfr	5,10-methylenetetrahydrofolate reductase	0.49	0.0
Mr1	major histocompatibility complex, class I-related	0.49	0.0
Traf5	TNF receptor-associated factor 5	0.49	0.0
6720401G13Rik	RIKEN cDNA 6720401G13 gene	0.49	0.0
Wwc2	WW, C2 and coiled-coil domain containing 2	0.49	7.8
Zfp658	zinc finger protein 658	0.50	7.8
Actb	actin, beta	0.50	0.0
Hist1h4a /// Hist1h4b /// Hist1h4c /// Hist1h4d /// Hist1h4f /// Hist1h4h /// Hist1h4i /// Hist1h4j /// Hist1h4k /// Hist1h4m /// Hist1h4n /// Hist2h4 /// Hist4h4 /// LOC100862646	histone cluster 1, H4a /// histone cluster 1, H4b /// histone cluster 1, H4c /// histone cluster 1, H4d /// histone cluster 1, H4f /// histone cluster 1, H4h /// histone cluster 1, H4i /// histone cluster 1, H4j /// histone cluster 1, H4k /// histone cluster 1, H4m /// histone cluster 1, H4n /// histone cluster 2, H4 /// histone cluster 4, H4 /// histone H4-like	0.50	2.8
Plekha6	pleckstrin homology domain containing, family A member 6	0.50	0.0
Trpc2	transient receptor potential cation channel, subfamily C, member 2	0.50	0.0
Fam13c	family with sequence similarity 13, member C	0.50	0.0
Prkd2	protein kinase D2	0.50	0.0
Mtmr7	myotubularin related protein 7	0.50	2.8
Sult1d1	sulfotransferase family 1D, member 1	0.50	2.8

2810416A17Rik	RIKEN cDNA 2810416A17 gene	0.50	2.8
Hyal2	hyaluronoglucosaminidase 2	0.50	0.0
Actc1	actin, alpha, cardiac muscle 1	0.50	2.8
Mtap4	Microtubule-associated protein 4	0.50	7.8
Tcf19	transcription factor 19	0.50	0.0
Banp	BTG3 associated nuclear protein	0.50	0.0
Pcmtd2	protein-L-isoaspartate (D-aspartate) O-methyltransferase domain containing 2	0.50	0.0
Odf3b	outer dense fiber of sperm tails 3B	0.50	0.0
Abhd14b	abhydrolase domain containing 14b	0.50	0.0
1700007K13Rik	RIKEN cDNA 1700007K13 gene	0.50	0.0
Mmab	methylmalonic aciduria (cobalamin deficiency) type B homolog (human)	0.50	0.0
Hes6	hairy and enhancer of split 6 (Drosophila)	0.50	0.0
Snx21	sorting nexin family member 21	0.50	0.0
Nthl1	nth (endonuclease III)-like 1 (E.coli)	0.50	2.8
Pds5b	PDS5, regulator of cohesion maintenance, homolog B (S. cerevisiae)	0.50	0.0
Ptgr2	prostaglandin reductase 2	0.50	0.0
Eif4e2	eukaryotic translation initiation factor 4E member 2	2.00	0.0
Wac	WW domain containing adaptor with coiled-coil	2.00	0.0
8030448K23Rik	RIKEN cDNA 8030448K23 gene	2.00	0.0
Kcnk1	potassium channel, subfamily K, member 1	2.00	0.0
2010002N04Rik	RIKEN cDNA 2010002N04 gene	2.00	0.0
Plxna2	plexin A2	2.00	0.0
Rps20	ribosomal protein S20	2.00	0.0
LOC269472	uncharacterized LOC269472	2.00	0.0
AU021128	expressed sequence AU021128	2.01	5.2
Usp53	ubiquitin specific peptidase 53	2.01	5.2
Plscr1	phospholipid scramblase 1	2.01	0.0
St6galnac4	ST6 (alpha-N-acetyl-neuraminyl-2,3-beta-galactosyl-1,3)-N-acetylgalactosaminide alpha-2,6-sialyltransferase 4	2.01	0.0
Ndrg1	N-myc downstream regulated gene 1	2.01	0.0
Dusp16	dual specificity phosphatase 16	2.01	0.0
Edn2	endothelin 2	2.01	0.0
Pim1	proviral integration site 1	2.01	0.0
Map2k3	mitogen-activated protein kinase kinase 3	2.01	0.0
Cdk13	cyclin-dependent kinase 13	2.01	0.0
Gm5620 /// Gm6682 /// LOC100862648 /// Tuba1a /// Tuba1b /// Tuba1c	predicted gene 5620 /// tubulin, alpha 1C pseudogene /// tubulin alpha-1C chain-like /// tubulin, alpha 1A /// tubulin, alpha 1B /// tubulin, alpha 1C	2.01	0.0
Padi4	peptidyl arginine deiminase, type IV	2.02	0.0
Nipal1	NIPA-like domain containing 1	2.02	5.2
Fam188a	family with sequence similarity 188, member A	2.02	0.0
Klhl15	kelch-like 15 (Drosophila)	2.02	0.0
Gcnt2	glucosaminyl (N-acetyl) transferase 2, I-branching enzyme	2.02	0.0
Sesn1	sestrin 1	2.02	0.0
2310058N22Rik	RIKEN cDNA 2310058N22 gene	2.02	0.0

Mboat1	membrane bound O-acyltransferase domain containing 1	2.03	0.0
Nop58	NOP58 ribonucleoprotein homolog (yeast)	2.03	0.0
Ept1	ethanolaminephosphotransferase 1 (CDP-ethanolamine-specific)	2.03	5.2
Rpf1	ribosome production factor 1 homolog (S. cerevisiae)	2.03	0.0
Cd84	CD84 antigen	2.03	0.0
2510009E07Rik	RIKEN cDNA 2510009E07 gene	2.04	0.0
1810032O08Rik	RIKEN cDNA 1810032O08 gene	2.04	0.0
Sco1	SCO cytochrome oxidase deficient homolog 1 (yeast)	2.04	0.0
Zwint	ZW10 interactor	2.04	0.0
Rpap2	RNA polymerase II associated protein 2	2.05	0.0
Brd7	bromodomain containing 7	2.05	0.0
Rbl1	retinoblastoma-like 1 (p107)	2.05	0.0
Abcc4	ATP-binding cassette, sub-family C (CFTR/MRP), member 4	2.05	0.0
LOC100862515 /// Tbc1d1	TBC1 domain family member 1-like /// TBC1 domain family, member 1	2.05	0.0
Pappa	pregnancy-associated plasma protein A	2.06	0.0
D3Ertd740e	DNA segment, Chr 3, ERATO Doi 740, expressed	2.06	0.0
Tlr4	toll-like receptor 4	2.06	0.0
8430408G22Rik	RIKEN cDNA 8430408G22 gene	2.06	0.0
Pabpc1l	poly(A) binding protein, cytoplasmic 1-like	2.06	0.0
Rai14	Retinoic acid induced 14	2.06	0.0
Tnfaip3	tumor necrosis factor, alpha-induced protein 3	2.07	0.0
Tmem120a	transmembrane protein 120A	2.07	0.0
Fhl2	four and a half LIM domains 2	2.07	0.0
Copa	coatomer protein complex subunit alpha	2.07	0.0
Rhoc	ras homolog gene family, member C	2.07	0.0
1700071A11Rik	RIKEN cDNA 1700071A11 gene	2.07	0.0
Ctse	cathepsin E	2.07	5.2
Slc9a8	solute carrier family 9 (sodium/hydrogen exchanger), member 8	2.07	0.0
Pip5k1b	phosphatidylinositol-4-phosphate 5-kinase, type 1 beta	2.07	0.0
Ammecr1l	AMME chromosomal region gene 1-like	2.08	5.2
St3gal1	ST3 beta-galactoside alpha-2,3-sialyltransferase 1	2.08	0.0
Tm4sf1	transmembrane 4 superfamily member 1	2.08	0.0
Cdkn2b	cyclin-dependent kinase inhibitor 2B (p15, inhibits CDK4)	2.08	0.0
Pxmp3	Peroxisomal membrane protein 3	2.08	0.0
Cul2	cullin 2	2.09	0.0
Rbm39	RNA binding motif protein 39	2.09	0.0
Clptm1	cleft lip and palate associated transmembrane protein 1	2.09	0.0
Clip4	CAP-GLY domain containing linker protein family, member 4	2.09	5.2
Slc2a1	solute carrier family 2 (facilitated glucose transporter), member 1	2.10	0.0
Enc1	ectodermal-neural cortex 1	2.10	0.0
9430016H08Rik	RIKEN cDNA 9430016H08 gene	2.10	0.0
Orc4	origin recognition complex, subunit 4	2.10	0.0
Pik3r5	phosphoinositide-3-kinase, regulatory subunit 5, p101	2.10	0.0

Cdc42	cell division cycle 42	2.10	0.0
B930098A02Rik /// Chic2	RIKEN cDNA B930098A02 gene /// cysteine-rich hydrophobic domain 2	2.11	0.0
Them5	thioesterase superfamily member 5	2.11	0.0
Zfp800	zinc finger protein 800	2.11	0.0
Itgam	integrin alpha M	2.12	5.2
Nus1	nuclear undecaprenyl pyrophosphate synthase 1 homolog (S. cerevisiae)	2.12	0.0
AU014973	expressed sequence AU014973	2.12	0.0
Fstl3	follistatin-like 3	2.12	0.0
Ptprk	protein tyrosine phosphatase, receptor type, K	2.13	0.0
Wdr61	WD repeat domain 61	2.13	0.0
Star	steroidogenic acute regulatory protein	2.13	0.0
Malt1	mucosa associated lymphoid tissue lymphoma translocation gene 1	2.14	0.0
Cdk9	cyclin-dependent kinase 9 (CDC2-related kinase)	2.14	0.0
Lmnb1	lamin B1	2.14	0.0
Grwd1	glutamate-rich WD repeat containing 1	2.14	0.0
E030047P09Rik	RIKEN cDNA E030047P09 gene	2.14	0.0
9830001H06Rik	RIKEN cDNA 9830001H06 gene	2.14	5.2
Tgm1	transglutaminase 1, K polypeptide	2.14	0.0
Myh9	myosin, heavy polypeptide 9, non-muscle	2.15	0.0
Sla	src-like adaptor	2.15	0.0
4833412E19Rik	RIKEN cDNA 4833412E19 gene	2.15	0.0
LOC100044742 /// Ptp4a1	protein tyrosine phosphatase type IVA 1-like /// protein tyrosine phosphatase 4a1	2.15	0.0
Adamts1	a disintegrin-like and metallopeptidase (reprolysin type) with thrombospondin type 1 motif, 1	2.15	0.0
March3	membrane-associated ring finger (C3HC4) 3	2.15	0.0
Bmp2k	BMP2 inducible kinase	2.15	0.0
Adarb1	adenosine deaminase, RNA-specific, B1	2.16	0.0
Pmepa1	prostate transmembrane protein, androgen induced 1	2.16	0.0
Mex3c	mex3 homolog C (C. elegans)	2.16	0.0
Klrb1b	killer cell lectin-like receptor subfamily B member 1B	2.16	0.0
Sdc4	syndecan 4	2.16	0.0
Phf3	PHD finger protein 3	2.17	0.0
Csf2rb2	colony stimulating factor 2 receptor, beta 2, low-affinity (granulocyte-macrophage)	2.17	0.0
D10Ertd709e	DNA segment, Chr 10, ERATO Doi 709, expressed	2.18	0.0
Gas5 /// Snord47	growth arrest specific 5 /// small nucleolar RNA, C/D box 47	2.18	0.0
Cxcr7	chemokine (C-X-C motif) receptor 7	2.18	0.0
Mpzl3	myelin protein zero-like 3	2.18	0.0
Rps17	ribosomal protein S17	2.18	0.0
Adamtsl2	ADAMTS-like 2	2.18	0.0
Zfp259	zinc finger protein 259	2.18	0.0
Camk2d	calcium/calmodulin-dependent protein kinase II, delta	2.19	0.0
Snx18	sorting nexin 18	2.20	0.0
Esd /// Gm2904	esterase D/formylglutathione hydrolase /// predicted pseudogene 2904	2.20	0.0

Itga5	integrin alpha 5 (fibronectin receptor alpha)	2.20	0.0
Dfna5	deafness, autosomal dominant 5 (human)	2.20	0.0
1700012B15Rik	RIKEN cDNA 1700012B15 gene	2.20	0.0
Trim16	tripartite motif-containing 16	2.20	0.0
Map2k7	mitogen-activated protein kinase kinase 7	2.21	5.2
5830407E08Rik	RIKEN cDNA 5830407E08 gene	2.21	0.0
Fosl2	fos-like antigen 2	2.22	0.0
Kdelr3	KDEL (Lys-Asp-Glu-Leu) endoplasmic reticulum protein retention receptor 3	2.23	0.0
Camk2n2	calcium/calmodulin-dependent protein kinase II inhibitor 2	2.23	0.0
Dot1l	DOT1-like, histone H3 methyltransferase (S. cerevisiae)	2.23	0.0
Tpcn2	two pore segment channel 2	2.23	0.0
Nes	nestin	2.23	0.0
F2rl1	coagulation factor II (thrombin) receptor-like 1	2.23	0.0
Mbp	myelin basic protein	2.23	0.0
5830416P10Rik	RIKEN cDNA 5830416P10 gene	2.23	0.0
Lnx2	ligand of numb-protein X 2	2.24	0.0
Dusp8	dual specificity phosphatase 8	2.24	0.0
Ipcef1	interaction protein for cytohesin exchange factors 1	2.24	0.0
Slc7a1	solute carrier family 7 (cationic amino acid transporter, y+ system), member 1	2.24	0.0
Gnpnat1	glucosamine-phosphate N-acetyltransferase 1	2.25	0.0
5430434G16Rik	RIKEN cDNA 5430434G16 gene	2.25	0.0
Tnfrsf22 /// Tnfrsf23	tumor necrosis factor receptor superfamily, member 22 /// tumor necrosis factor receptor superfamily, member 23	2.25	0.0
Fam122a	family with sequence similarity 122, member A	2.25	0.0
Loxl1	lysyl oxidase-like 1	2.25	0.0
2900046L07Rik	RIKEN cDNA 2900046L07 gene	2.25	5.2
4833441D16Rik	RIKEN cDNA 4833441D16 gene	2.25	0.0
Lass3	LAG1 homolog, ceramide synthase 3	2.26	0.0
Cidea	cell death-inducing DNA fragmentation factor, alpha subunit-like effector A	2.26	0.0
Epha2	Eph receptor A2	2.27	0.0
Artn	artemin	2.27	0.0
Uck2	uridine-cytidine kinase 2	2.27	0.0
Il17ra	interleukin 17 receptor A	2.29	0.0
Sowahb	sosondowah ankyrin repeat domain family member B	2.29	0.0
Sh3bp2	SH3-domain binding protein 2	2.29	0.0
Alyref	Aly/REF export factor	2.29	0.0
Tes	testis derived transcript	2.29	0.0
Nuak2	NUAK family, SNF1-like kinase, 2	2.29	0.0
Slc25a25	solute carrier family 25 (mitochondrial carrier, phosphate carrier), member 25	2.29	0.0
AU019559	expressed sequence AU019559	2.30	0.0
Afp	alpha fetoprotein	2.30	0.0
C1d	C1D nuclear receptor co-repressor	2.30	0.0
Ugcg	UDP-glucose ceramide glucosyltransferase	2.31	0.0
Trim24	tripartite motif-containing 24	2.31	5.2
Eif2ak1	eukaryotic translation initiation factor 2 alpha kinase 1	2.31	0.0

Lrrc8c	leucine rich repeat containing 8 family, member C	2.31	0.0
B230303O12Rik	RIKEN cDNA B230303O12 gene	2.31	0.0
Tubb2b	tubulin, beta 2B class IIB	2.32	0.0
Ptger4	prostaglandin E receptor 4 (subtype EP4)	2.32	0.0
6720477C19Rik	RIKEN cDNA 6720477C19 gene	2.32	0.0
Ppargc1a	peroxisome proliferative activated receptor, gamma, coactivator 1 alpha	2.33	0.0
Gimap6	GTPase, IMAP family member 6	2.33	0.0
Adipor1	adiponectin receptor 1	2.33	0.0
Gda	guanine deaminase	2.34	0.0
Zc3hav1	zinc finger CCCH type, antiviral 1	2.34	0.0
Trp53inp2	Transformation related protein 53 inducible nuclear protein 2	2.34	0.0
Sdad1	SDA1 domain containing 1	2.35	0.0
Tmcc3	transmembrane and coiled coil domains 3	2.35	0.0
Prrc1	proline-rich coiled-coil 1	2.36	0.0
Ms4a6b	membrane-spanning 4-domains, subfamily A, member 6B	2.36	0.0
Picalm	phosphatidylinositol binding clathrin assembly protein	2.36	0.0
Csf2rb	colony stimulating factor 2 receptor, beta, low-affinity (granulocyte-macrophage)	2.36	0.0
Dnajb5	DnaJ (Hsp40) homolog, subfamily B, member 5	2.36	0.0
Samsn1	SAM domain, SH3 domain and nuclear localization signals, 1	2.36	5.2
Prosapip1	ProSAPiP1 protein	2.36	0.0
C80120	expressed sequence C80120	2.37	0.0
Mbd1	methyl-CpG binding domain protein 1	2.37	0.0
A330106F07Rik	RIKEN cDNA A330106F07 gene	2.37	5.2
Irs2	insulin receptor substrate 2	2.37	0.0
Gpr65	G-protein coupled receptor 65	2.37	0.0
Col5a1	collagen, type V, alpha 1	2.38	0.0
Srsf7	serine/arginine-rich splicing factor 7	2.39	0.0
E030010A14Rik	RIKEN cDNA E030010A14 gene	2.39	0.0
Shb	src homology 2 domain-containing transforming protein B	2.39	0.0
B230216G23Rik	RIKEN cDNA B230216G23 gene	2.39	0.0
Ttc9	tetratricopeptide repeat domain 9	2.39	0.0
Srf	serum response factor	2.39	0.0
Zfml	Zinc finger, matrin-like	2.39	0.0
Prg4	proteoglycan 4 (megakaryocyte stimulating factor, articular superficial zone protein)	2.39	0.0
Hbegf	heparin-binding EGF-like growth factor	2.40	0.0
Smox	spermine oxidase	2.40	0.0
Rps9	ribosomal protein S9	2.40	0.0
Mpp6	membrane protein, palmitoylated 6 (MAGUK p55 subfamily member 6)	2.40	0.0
Sema4c	sema domain, immunoglobulin domain (Ig), transmembrane domain (TM) and short cytoplasmic domain, (semaphorin) 4C	2.41	0.0
Egr2	early growth response 2	2.41	0.0
Daam2	dishevelled associated activator of morphogenesis 2	2.41	0.0
Smpd4	sphingomyelin phosphodiesterase 4	2.41	0.0

Noc2l	nucleolar complex associated 2 homolog (S. cerevisiae)	2.41	0.0
Spsb1	splA/ryanodine receptor domain and SOCS box containing 1	2.41	5.2
Vcan	versican	2.43	0.0
Upp1	uridine phosphorylase 1	2.43	5.2
Tmem108	transmembrane protein 108	2.44	0.0
Heyl	hairy/enhancer-of-split related with YRPW motif-like	2.44	0.0
Tacc3	transforming, acidic coiled-coil containing protein 3	2.44	0.0
Clec4n	C-type lectin domain family 4, member n	2.45	0.0
1700017B05Rik	RIKEN cDNA 1700017B05 gene	2.45	0.0
Itpkc	inositol 1,4,5-trisphosphate 3-kinase C	2.45	0.0
Itgb6	integrin beta 6	2.45	0.0
Dbn1	drebrin 1	2.46	0.0
Col3a1	collagen, type III, alpha 1	2.46	0.0
Syncrip	synaptotagmin binding, cytoplasmic RNA interacting protein	2.46	0.0
Mapk6	mitogen-activated protein kinase 6	2.46	0.0
Zkscan5	zinc finger with KRAB and SCAN domains 5	2.46	0.0
Prmt8	protein arginine N-methyltransferase 8	2.47	0.0
Arl4c	ADP-ribosylation factor-like 4C	2.47	0.0
Rnd3	Rho family GTPase 3	2.48	0.0
Sorbs1	sorbin and SH3 domain containing 1	2.48	0.0
Timp1	tissue inhibitor of metalloproteinase 1	2.48	0.0
Kcnk5	potassium channel, subfamily K, member 5	2.49	0.0
Plat	plasminogen activator, tissue	2.49	0.0
Nid1	nidogen 1	2.49	0.0
F3	coagulation factor III	2.50	0.0
Pla2g7	phospholipase A2, group VII (platelet-activating factor acetylhydrolase, plasma)	2.50	0.0
Neurl3	neuralized homolog 3 homolog (Drosophila)	2.50	0.0
Fam176a	family with sequence similarity 176, member A	2.50	0.0
March1	membrane-associated ring finger (C3HC4) 1	2.50	0.0
Rasl11b	RAS-like, family 11, member B	2.51	0.0
AI607873	expressed sequence AI607873	2.51	0.0
D8Ertd82e	DNA segment, Chr 8, ERATO Doi 82, expressed	2.52	0.0
Entpd4 /// LOC100862375	ectonucleoside triphosphate diphosphohydrolase 4 /// ectonucleoside triphosphate diphosphohydrolase 4-like	2.52	0.0
Rnf152	ring finger protein 152	2.52	0.0
Egfr	epidermal growth factor receptor	2.53	0.0
Btg1	B cell translocation gene 1, anti-proliferative	2.53	0.0
Gm8369	predicted gene 8369	2.53	0.0
Gm11425 /// LOC630855 /// Rpl12	predicted gene 11425 /// 60S ribosomal protein L12-like /// ribosomal protein L12	2.54	0.0
Mfsd2b	Major facilitator superfamily domain containing 2B	2.54	0.0
Unc5b	unc-5 homolog B (C. elegans)	2.55	0.0
Bcar1	breast cancer anti-estrogen resistance 1	2.55	0.0
Krt18	keratin 18	2.56	0.0
Gfpt2	glutamine fructose-6-phosphate transaminase 2	2.56	0.0
Kalrn	kalirin, RhoGEF kinase	2.56	5.2
9930012K11Rik	RIKEN cDNA 9930012K11 gene	2.56	0.0

2310014L17Rik	RIKEN cDNA 2310014L17 gene	2.56	0.0
Twistnb	TWIST neighbor	2.56	0.0
Gadd45a	growth arrest and DNA-damage-inducible 45 alpha	2.56	0.0
Nedd9	neural precursor cell expressed, developmentally down-regulated gene 9	2.57	0.0
Snx10	Sorting nexin 10	2.57	0.0
Itk	IL2 inducible T cell kinase	2.57	0.0
Lrp8	low density lipoprotein receptor-related protein 8, apolipoprotein e receptor	2.58	0.0
Eprs	glutamyl-prolyl-tRNA synthetase	2.58	0.0
C230007H23Rik	RIKEN cDNA C230007H23 gene	2.58	0.0
Gna13	guanine nucleotide binding protein, alpha 13	2.59	0.0
Crem	cAMP responsive element modulator	2.59	0.0
4833411I10Rik	RIKEN cDNA 4833411I10 gene	2.60	0.0
Gp49a /// Lilrb4	glycoprotein 49 A /// leukocyte immunoglobulin-like receptor, subfamily B, member 4	2.61	0.0
Slc20a1	solute carrier family 20, member 1	2.61	0.0
E430014B02Rik	RIKEN cDNA E430014B02 gene	2.61	0.0
Uchl1	ubiquitin carboxy-terminal hydrolase L1	2.63	0.0
Klhl29	kelch-like 29 (Drosophila)	2.64	0.0
Prdm2	PR domain containing 2, with ZNF domain	2.64	0.0
Gm3776 /// Gsta1 /// Gsta2	predicted gene 3776 /// glutathione S-transferase, alpha 1 (Ya) /// glutathione S-transferase, alpha 2 (Yc2)	2.64	0.0
Gas2l3	growth arrest-specific 2 like 3	2.65	0.0
Tacstd2	tumor-associated calcium signal transducer 2	2.65	0.0
AA408650	expressed sequence AA408650	2.65	0.0
Zbtb20	zinc finger and BTB domain containing 20	2.66	0.0
Nfkbid	nuclear factor of kappa light polypeptide gene enhancer in B cells inhibitor, delta	2.66	0.0
Glipr1	GLI pathogenesis-related 1 (glioma)	2.66	0.0
Psme4	proteasome (prosome, macropain) activator subunit 4	2.67	0.0
5730559C18Rik	RIKEN cDNA 5730559C18 gene	2.67	0.0
Arg2	arginase type II	2.67	0.0
Ccdc86	coiled-coil domain containing 86	2.68	0.0
Adamts2	a disintegrin-like and metallopeptidase (reprolysin type) with thrombospondin type 1 motif, 2	2.69	0.0
2700038G22Rik	RIKEN cDNA 2700038G22 gene	2.70	0.0
Preb	prolactin regulatory element binding	2.70	0.0
Itprip	inositol 1,4,5-triphosphate receptor interacting protein	2.71	0.0
Rin1	Ras and Rab interactor 1	2.71	0.0
Flnb	filamin, beta	2.71	0.0
Btla	B and T lymphocyte associated	2.72	0.0
C230085N15Rik	RIKEN cDNA C230085N15 gene	2.72	0.0
Krt8	keratin 8	2.72	0.0
Fem1c	fem-1 homolog c (C.elegans)	2.73	0.0
Gpr171	G protein-coupled receptor 171	2.74	0.0
Chd1	chromodomain helicase DNA binding protein 1	2.74	0.0
Peg3	paternally expressed 3	2.74	0.0
Pvr	poliovirus receptor	2.75	0.0
4833422B07Rik	RIKEN cDNA 4833422B07 gene	2.75	0.0

Rnf144b	ring finger protein 144B	2.76	5.2
Slc7a6	solute carrier family 7 (cationic amino acid transporter, y+ system), member 6	2.77	0.0
4930523C07Rik	RIKEN cDNA 4930523C07 gene	2.78	0.0
Nudt6	nudix (nucleoside diphosphate linked moiety X)-type motif 6	2.78	0.0
Plcxd2	phosphatidylinositol-specific phospholipase C, X domain containing 2	2.78	0.0
Srxn1	sulfiredoxin 1 homolog (S. cerevisiae)	2.79	5.2
Trib1	tribbles homolog 1 (Drosophila)	2.79	0.0
Myo1e	myosin IE	2.79	0.0
Tox	thymocyte selection-associated high mobility group box	2.79	0.0
Litaf	LPS-induced TN factor	2.81	0.0
Sphk1	sphingosine kinase 1	2.81	0.0
Plk3	polo-like kinase 3	2.81	0.0
Cd28	CD28 antigen	2.82	0.0
Anxa1	annexin A1	2.83	5.2
Gprc5b	G protein-coupled receptor, family C, group 5, member B	2.83	0.0
Lox	lysyl oxidase	2.84	0.0
Zfand5	zinc finger, AN1-type domain 5	2.85	0.0
Pkib	protein kinase inhibitor beta, cAMP dependent, testis specific	2.87	0.0
Dnajc25	DnaJ (Hsp40) homolog, subfamily C, member 25	2.92	0.0
Armcx5	armadillo repeat containing, X-linked 5	2.92	0.0
Slc22a3	solute carrier family 22 (organic cation transporter), member 3	2.93	0.0
Rab20	RAB20, member RAS oncogene family	2.93	0.0
Gab2	growth factor receptor bound protein 2-associated protein 2	2.95	0.0
Sh3pxd2b	SH3 and PX domains 2B	2.95	0.0
LOC100862072 /// Loxl2	lysyl oxidase homolog 2-like /// lysyl oxidase-like 2	2.97	0.0
Fam46b	family with sequence similarity 46, member B	2.97	0.0
Slc7a11	solute carrier family 7 (cationic amino acid transporter, y+ system), member 11	2.97	5.2
Slc12a9	solute carrier family 12 (potassium/chloride transporters), member 9	2.98	0.0
Ptpn22	protein tyrosine phosphatase, non-receptor type 22 (lymphoid)	3.00	0.0
Fcgr4	Fc receptor, IgG, low affinity IV	3.01	0.0
Col1a1	collagen, type I, alpha 1	3.01	0.0
Elp4	elongation protein 4 homolog (S. cerevisiae)	3.01	0.0
Pdk4	pyruvate dehydrogenase kinase, isoenzyme 4	3.01	0.0
Eln	elastin	3.02	0.0
Tlr13	toll-like receptor 13	3.02	0.0
Slco1c1	solute carrier organic anion transporter family, member 1c1	3.02	0.0
Id2	inhibitor of DNA binding 2	3.02	0.0
Cd300lf	CD300 antigen like family member F	3.04	0.0
2810403A07Rik	RIKEN cDNA 2810403A07 gene	3.05	0.0
G530011O06Rik	RIKEN cDNA G530011O06 gene	3.06	0.0

Zfp106	zinc finger protein 106	3.07	0.0
Leo1	Leo1, Paf1/RNA polymerase II complex component, homolog (S. cerevisiae)	3.07	0.0
Slc39a14	solute carrier family 39 (zinc transporter), member 14	3.08	0.0
Inhbb	inhibin beta-B	3.09	0.0
D14Ertd449e /// Gm10395 /// Gm9746	DNA segment, Chr 14, ERATO Doi 449, expressed /// predicted gene 10395 /// predicted gene 9746	3.10	0.0
Chd7	chromodomain helicase DNA binding protein 7	3.10	0.0
Thbs1	thrombospondin 1	3.11	0.0
Dusp3	dual specificity phosphatase 3 (vaccinia virus phosphatase VH1-related)	3.13	0.0
Ikzf4	IKAROS family zinc finger 4	3.14	0.0
Tnc	tenascin C	3.14	0.0
9130221J17Rik	RIKEN cDNA 9130221J17 gene	3.16	0.0
Rcan1	regulator of calcineurin 1	3.16	0.0
5830453J16Rik	RIKEN cDNA 5830453J16 gene	3.16	0.0
Pdlim7	PDZ and LIM domain 7	3.17	0.0
Stc1	stanniocalcin 1	3.19	0.0
Rprd2	regulation of nuclear pre-mRNA domain containing 2	3.20	0.0
Cdr2	cerebellar degeneration-related 2	3.21	0.0
9630009C16	uncharacterized protein 9630009C16	3.21	0.0
Plk2	polo-like kinase 2	3.22	0.0
Mrpl15	mitochondrial ribosomal protein L15	3.24	0.0
Rhou	ras homolog gene family, member U	3.25	0.0
Hk2	hexokinase 2	3.27	0.0
Plaur	plasminogen activator, urokinase receptor	3.32	0.0
Il1rn	interleukin 1 receptor antagonist	3.32	0.0
Mbnl3	muscleblind-like 3 (Drosophila)	3.35	0.0
2010109K09Rik	RIKEN cDNA 2010109K09 gene	3.37	0.0
5033430I15Rik	RIKEN cDNA 5033430I15 gene	3.37	0.0
Cald1	caldesmon 1	3.38	0.0
Akap12	A kinase (PRKA) anchor protein (gravin) 12	3.39	0.0
Abtb2	ankyrin repeat and BTB (POZ) domain containing 2	3.42	0.0
Cflar	CASP8 and FADD-like apoptosis regulator	3.44	0.0
Gjb2	gap junction protein, beta 2	3.45	0.0
Sdcbp2	syndecan binding protein (syntenin) 2	3.47	0.0
Eppk1	epiplakin 1	3.48	0.0
Rbm12	RNA binding motif protein 12	3.48	0.0
Pi15	peptidase inhibitor 15	3.48	0.0
Cxcl1	chemokine (C-X-C motif) ligand 1	3.48	0.0
Spon2	spondin 2, extracellular matrix protein	3.51	0.0
1700109H08Rik	RIKEN cDNA 1700109H08 gene	3.51	0.0
Rasd1	RAS, dexamethasone-induced 1	3.53	0.0
Adamts15	a disintegrin-like and metallopeptidase (reprolysin type) with thrombospondin type 1 motif, 15	3.54	0.0
Slfn1	schlafen 1	3.55	5.2
Slc16a6	solute carrier family 16 (monocarboxylic acid transporters), member 6	3.58	0.0
Selp	selectin, platelet	3.58	0.0
Ptpn2	protein tyrosine phosphatase, non-receptor type 2	3.61	0.0

Spry4	sprouty homolog 4 (Drosophila)	3.63	0.0
Cdkn1a	cyclin-dependent kinase inhibitor 1A (P21)	3.64	0.0
6330406I15Rik	RIKEN cDNA 6330406I15 gene	3.65	0.0
Maff	v-maf musculoaponeurotic fibrosarcoma oncogene family, protein F (avian)	3.65	0.0
Syk	spleen tyrosine kinase	3.66	0.0
H2-Q10	histocompatibility 2, Q region locus 10	3.66	0.0
Gadd45g	growth arrest and DNA-damage-inducible 45 gamma	3.66	0.0
Slc16a13	solute carrier family 16 (monocarboxylic acid transporters), member 13	3.68	0.0
9430076C15Rik	RIKEN cDNA 9430076C15 gene	3.68	0.0
Pmvk	phosphomevalonate kinase	3.69	0.0
Slc38a4	solute carrier family 38, member 4	3.74	0.0
Il18rap	interleukin 18 receptor accessory protein	3.74	0.0
Ccr1	chemokine (C-C motif) receptor 1	3.77	0.0
Sbno2	strawberry notch homolog 2 (Drosophila)	3.82	0.0
Socs3	suppressor of cytokine signaling 3	3.84	0.0
Btbd11	BTB (POZ) domain containing 11	3.88	0.0
Bcl3	B cell leukemia/lymphoma 3	3.89	0.0
Gm19966	predicted gene, 19966	3.94	0.0
Nlrc3	NLR family, CARD domain containing 3	3.95	0.0
Sgms2	sphingomyelin synthase 2	4.01	0.0
Tnfrsf12a	tumor necrosis factor receptor superfamily, member 12a	4.01	0.0
O3far1	omega-3 fatty acid receptor 1	4.01	0.0
Prkcg	protein kinase C, gamma	4.03	0.0
Tnfaip6	tumor necrosis factor alpha induced protein 6	4.04	0.0
Adamts4	a disintegrin-like and metallopeptidase (reprolysin type) with thrombospondin type 1 motif, 4	4.06	0.0
Tubb6	tubulin, beta 6 class V	4.06	0.0
Dus4l	dihydrouridine synthase 4-like (S. cerevisiae)	4.09	0.0
Gm3893 /// Gm5859 /// Gm7819 /// LOC100861635 /// LOC100862237	predicted gene 3893 /// predicted pseudogene 5859 /// predicted gene 7819 /// protein FAM205A-like /// uncharacterized LOC100862237	4.10	0.0
Mt1	metallothionein 1	4.10	0.0
Cldn23	claudin 23	4.11	0.0
Obfc2a	oligonucleotide/oligosaccharide-binding fold containing 2A	4.14	0.0
9230110K08Rik	RIKEN cDNA 9230110K08 gene	4.18	0.0
Ereg	epiregulin	4.19	0.0
Acta1	actin, alpha 1, skeletal muscle	4.21	0.0
Gem	GTP binding protein (gene overexpressed in skeletal muscle)	4.27	0.0
Trem1	triggering receptor expressed on myeloid cells 1	4.29	0.0
Bcl6b	B cell CLL/lymphoma 6, member B	4.37	0.0
Serpine1	serine (or cysteine) peptidase inhibitor, clade E, member 1	4.38	0.0
Slc26a4	solute carrier family 26, member 4	4.39	0.0
Sprr1a	small proline-rich protein 1A	4.51	0.0
Cd69	CD69 antigen	4.56	0.0
Tmem242	transmembrane protein 242	4.60	0.0

Pde10a	phosphodiesterase 10A	4.61	0.0
Itpr2	Inositol 1,4,5-triphosphate receptor 2	4.62	0.0
Txndc9	thioredoxin domain containing 9	4.63	0.0
Myc	myelocytomatosis oncogene	4.64	0.0
Adm	adrenomedullin	4.66	0.0
Kcne4	potassium voltage-gated channel, Isk-related subfamily, gene 4	4.69	0.0
Adora2b	adenosine A2b receptor	4.71	0.0
Ccrn4l	CCR4 carbon catabolite repression 4-like (S. cerevisiae)	4.75	0.0
Marco	macrophage receptor with collagenous structure	4.81	0.0
Ppp2ca	protein phosphatase 2 (formerly 2A), catalytic subunit, alpha isoform	4.88	0.0
4933409K07Rik /// Gm10590 /// Gm2163 /// Gm3893 /// Gm5859 /// Gm7819 /// LOC100041516 /// LOC100861610 /// LOC100861635	RIKEN cDNA 4933409K07 gene /// predicted gene 10590 /// predicted gene 2163 /// predicted gene 3893 /// predicted pseudogene 5859 /// predicted gene 7819 /// protein FAM205A-like /// uncharacterized LOC100861610 /// protein FAM205A-like	4.89	0.0
Ly6g6c	lymphocyte antigen 6 complex, locus G6C	4.89	0.0
Cd33	CD33 antigen	5.01	0.0
Tlk2	tousled-like kinase 2 (Arabidopsis)	5.08	0.0
Adamts9	a disintegrin-like and metallopeptidase (reprolysin type) with thrombospondin type 1 motif, 9	5.13	0.0
Sdcbp	syndecan binding protein	5.25	0.0
Gdnf	glial cell line derived neurotrophic factor	5.27	0.0
D14Ertd449e	DNA segment, Chr 14, ERATO Doi 449, expressed	5.29	5.2
Mfsd2a	major facilitator superfamily domain containing 2A	5.32	0.0
Clec4d	C-type lectin domain family 4, member d	5.40	0.0
Cxcl5	chemokine (C-X-C motif) ligand 5	5.44	0.0
Gm13889	predicted gene 13889	5.52	0.0
AU023617	expressed sequence AU023617	5.54	0.0
Fbxw10 /// Trim16	F-box and WD-40 domain protein 10 /// tripartite motif-containing 16	5.71	0.0
Calca	calcitonin/calcitonin-related polypeptide, alpha	5.78	0.0
Mt2	metallothionein 2	5.81	0.0
Areg	amphiregulin	5.86	0.0
Iffo2	intermediate filament family orphan 2	5.97	0.0
Rdh12	retinol dehydrogenase 12	5.99	0.0
Nfe2l2	nuclear factor, erythroid derived 2, like 2	6.08	0.0
AA467197	expressed sequence AA467197	6.12	0.0
Trim15	tripartite motif-containing 15	6.31	0.0
Sfn	stratifin	7.04	0.0
Creb5	cAMP responsive element binding protein 5	7.12	0.0
Arhgef7	Rho guanine nucleotide exchange factor (GEF7)	7.13	0.0
Alpl	alkaline phosphatase, liver/bone/kidney	7.28	0.0
Arid5a	AT rich interactive domain 5A (MRF1-like)	8.25	0.0
Rad18	RAD18 homolog (S. cerevisiae)	8.28	0.0
Ptx3	pentraxin related gene	8.75	0.0
Clec4e	C-type lectin domain family 4, member e	8.95	0.0

Arntl	aryl hydrocarbon receptor nuclear translocator-like	9.95	0.0
Mmp8	matrix metallopeptidase 8	10.43	0.0
Apol8	apolipoprotein L 8	12.51	0.0
Fosl1	fos-like antigen 1	12.92	0.0
Prss22	protease, serine, 22	14.51	0.0
Angptl4	angiopoietin-like 4	15.94	0.0
Nfil3	nuclear factor, interleukin 3, regulated	18.51	0.0
Cxcl2	chemokine (C-X-C motif) ligand 2	19.14	0.0
Dio2	deiodinase, iodothyronine, type II	21.83	0.0
Has1	hyaluronan synthase1	22.59	0.0
Cldn4	claudin 4	27.05	0.0
Krtap17-1	keratin associated protein 17-1	29.21	0.0
Il1r2	interleukin 1 receptor, type II	29.39	0.0
Apold1	apolipoprotein L domain containing 1	39.16	0.0
Il6	interleukin 6	146.46	0.0

[a] FC: fold change, which is calculated by dividing the expression in VILI-exposed WT mice by the expression in WT control mice.
