# Supplementary material for: Genes Influenced by the Non-Muscle Isoform of Myosin Light Chain Kinase Impact Human Cancer Prognosis
Source: PLoS One. 2014 Apr 8;9(4):e94325. doi: 10.1371/journal.pone.0094325 (PMC3979809; doi:10.1371/journal.pone.0094325)
Supplement: Table S3 — Univariate Cox proportional hazards regression of overall survival against M38+MYLK signature status. (PDF) [file pone.0094325.s008.pdf]

Table S3. Univariate Cox proportional hazards regression of overall survival against M38+*MYLK* signature status

	Training			Testing		
Cancer	Hazard ratio	95% Confidence interval	*P*-value	Hazard ratio	95% Confidence interval	*P*-value
Breast	3.77	(1.91, 7.45)	< 0.001	2.65	(1.29, 5.45)	0.008
Colon	2.72	(1.66, 4.46)	< 0.001	2.93	(1.12, 7.65)	0.028
Glioma	2.62	(1.55, 4.43)	< 0.001	1.86	(0.92, 3.77)	0.085
Lung	2.68	(1.78, 4.04)	< 0.001	1.40	(0.93, 2.09)	0.106
